# Predictive ability of genomic selection models in a multi-population perennial ryegrass training set using genotyping-by-sequencing

**DOI:** 10.1007/s00122-017-3030-1

**Published:** 2017-12-20

**Authors:** Marty J. Faville, Siva Ganesh, Mingshu Cao, M. Z. Zulfi Jahufer, Timothy P. Bilton, H. Sydney Easton, Douglas L. Ryan, Jason A. K. Trethewey, M. Philip Rolston, Andrew G. Griffiths, Roger Moraga, Casey Flay, Jana Schmidt, Rachel Tan, Brent A. Barrett

**Affiliations:** 1 AgResearch Ltd, Grasslands Research Centre, PB 11008, Palmerston North, New Zealand; 2 AgResearch Ltd, Invermay Agricultural Centre, PB 50034, Mosgiel, New Zealand; 3 AgResearch Ltd, Ruakura Research Centre, PB 3123, Hamilton, New Zealand; 4 AgResearch Ltd, Lincoln Science Centre, PB 4749, Lincoln, New Zealand; 5 Present Address: PGG Wrightson Seeds Ltd, Ruakura Research Centre, Hamilton, New Zealand; 6 Present Address: Lincoln Agritech, PO Box 69 133, Lincoln, New Zealand

## Abstract

**Key message:**

Genomic prediction models for multi-year dry matter yield, via genotyping-by-sequencing in a composite training set, demonstrate potential for genetic gain improvement through within-half sibling family selection.

**Abstract:**

Perennial ryegrass (*Lolium perenne* L.) is a key source of nutrition for ruminant livestock in temperate environments worldwide. Higher seasonal and annual yield of herbage dry matter (DMY) is a principal breeding objective but the historical realised rate of genetic gain for DMY is modest. Genomic selection was investigated as a tool to enhance the rate of genetic gain. Genotyping-by-sequencing (GBS) was undertaken in a multi-population (MP) training set of five populations, phenotyped as half-sibling (HS) families in five environments over 2 years for mean herbage accumulation (HA), a measure of DMY potential. GBS using the ApeKI enzyme yielded 1.02 million single-nucleotide polymorphism (SNP) markers from a training set of *n* = 517. MP-based genomic prediction models for HA were effective in all five populations, cross-validation-predictive ability (PA) ranging from 0.07 to 0.43, by trait and target population, and 0.40–0.52 for days-to-heading. Best linear unbiased predictor (BLUP)-based prediction methods, including GBLUP with either a standard or a recently developed (KGD) relatedness estimation, were marginally superior or equal to ridge regression and random forest computational approaches. PA was principally an outcome of SNP modelling genetic relationships between training and validation sets, which may limit application for long-term genomic selection, due to PA decay. However, simulation using data from the training experiment indicated a twofold increase in genetic gain for HA, when applying a prediction model with moderate PA in a single selection cycle, by combining among-HS family selection, based on phenotype, with within-HS family selection using genomic prediction.

**Electronic supplementary material:**

The online version of this article (10.1007/s00122-017-3030-1) contains supplementary material, which is available to authorized users.

## Introduction

Genomic selection (GS), introduced by Meuwissen et al. (2001), describes breeding strategies in which the effects of many DNA markers throughout the genome are used to predict the breeding value (genomic-estimated breeding value, GEBV) of selection candidates. GS methodology has become widely implemented in animal breeding, particularly dairy cattle (Hayes et al. 2009a), and more recently theoretical and empirical assessments have been implemented for economic plant species (Heffner et al. 2009; Resende et al. 2012). GS has recently emerged as a prospect for forage plant species largely due to the advent of flexible, low-cost-single nucleotide polymorphism (SNP) marker platforms, such as genotyping-by-sequencing (GBS) (Elshire et al. 2011), that can provide the high-density marker information typically needed for GS (Annicchiarico et al. 2015; Fè et al. 2015a; Hayes et al. 2013).

Forage grasses and legumes are important plant species. Of the 38% of global land area under agricultural production (http://faostat3.fao.org), approximately 68% is used for pastoral agriculture. Pastures based on temperate forage species play a significant role within these agricultural systems with, for example, an estimated 80% of cow’s milk derived from temperate grassland agriculture (Wilkins and Humphreys 2003).

The temperate forage perennial ryegrass (*Lolium perenne* L.) (2*n* = 2*x* = 14) is the principal source of nutrition for ruminant livestock grazed in temperate-intensive pastoral agricultural systems, including New Zealand. Like most forage species, perennial ryegrass is obligately outcrossing and both natural and synthetic populations are genetically heterogeneous and characterised by high levels of heterozygosity (Sweeney and Danneberger 1994). Genetic improvement in ryegrass is commonly achieved by intra-population recurrent selection (Conaghan and Casler 2011) or systems based on phenotypic mass selection, supplemented by generation of new populations from inter-population crosses. Cultivars are typically synthetic populations generated by a random mating polycross amongst elite parent plants. Increased seasonal and annual level of herbage dry matter yield (DMY), a driver of livestock productivity, is a principal breeding objective for this species (Wilkins and Humphreys 2003; Williams et al. 2007; Chapman et al. 2017). However, the realised rate of DMY genetic gain has been modest, at between 3 and 4% per decade (Easton et al. 2002; Sampoux et al. 2010; Van Wijk and Reheul 1990). One underlying factor is that DMY is often not directly selected for in breeding programmes (Casler and Brummer 2008). While individual plant vigour or biomass may be routinely assessed in spaced plant nurseries, these traits have poor genetic correlation with DMY measured in a competitive sward (Hayward and Vivero 1984; Lazenby and Rogers 1964; Waldron et al. 2008).

Effective direct selection for DMY may best be achieved by assessment of half- or full-sibling family performance in seeded plots that correspond more closely to a sward (Annicchiarico et al. 2015), over a period of years and in more than one target environment to account for genotype-by-environment interaction (Conaghan et al. 2008; Jafari et al. 2003). This, however, has the consequence of increasing costs and lengthening the selection interval in a breeding programme, and therefore, the time taken to generate a candidate cultivar. This between-family selection approach also fails to get traction from the within-family genetic variance. If sufficiently accurate, indirect selection methods for DMY, such as marker-assisted selection (MAS) or GS, are of interest as they represent an opportunity to cost-efficiently select for a sward trait in single plants—enhancing accuracy of selection, shortening the breeding cycle (Heffner et al. 2010; Resende et al. 2014) and enabling application of selection pressure to within-family variation (Casler and Brummer 2008).

DMY and the majority of other agronomically important traits in forages are quantitative and highly polygenic (Dolstra et al. 2007; Wilkins and Humphreys 2003). Therefore, the genetic gain from QTL studies and MAS using one or a few QTL-linked markers is likely to be small, due to the low proportion of the total genetic variance captured (Heffner et al. 2009). GS has the capacity to address these limitations because (a) it can be applied directly in multi-parent breeding populations without prior QTL discovery in a separate research population; and (b) assuming a majority of contributing QTL are in LD with at least one marker locus, GS has the potential to capture the effects of most of the QTL governing a quantitative trait (Solberg et al. 2008) such as DMY. The potential of GS for improving genetic gain in forages has been addressed theoretically (Hayes et al. 2013; Resende et al. 2014) and now more recently through empirical investigation (Annicchiarico et al. 2015; Fè et al. 2015a, b; Grinberg et al. 2016; Li et al. 2015).

Our main objective was to develop and evaluate resources for GS for DMY and days-to-heading (DTH) in advanced perennial ryegrass populations in a commercial breeding programme, integrating multi-year phenotypic data from multiple locations, and including assessment of different data and statistical models for prediction. Our general approach was to construct genomic prediction models for parent plants using herbage accumulation (HA) data, as a measure of DMY potential, from sown plots of their half-sibling (HS) progenies. The literature on GS incorporates a wide-ranging selection of statistical models for determining GEBV’s. This includes GBLUP [genomic best linear unbiased prediction via a linear mixed model framework, using a genomic relationship matrix (GRM)] through to Bayesian, shrinkage and machine-learning alternatives (Crossa et al. 2016; Grinberg et al. 2016; Heslot et al. 2012). We utilised some of the most commonly used methods to estimate GEBV’s: (1) GBLUP with GRM estimated in two different ways, one being a novel statistical method (KGD) proposed by Dodds et al. (2015) for generating unbiased relatedness estimates from GBS SNP data; (2) ridge linear regression (RR; sometimes referred to as RR-BLUP); and (3) random forest regression (RF), a non-linear machine-learning method.

Furthermore, most plant studies to date regarding GS have considered a single-population scenario. Where individual population sizes are numerically small, combining phenotypes from multiple populations, analogous to multi-breed training sets in animals (de Roos et al. 2009; Schulz-Streeck et al. 2012; Porto-Neto et al. 2015), may be a way to increase the size of the training set and develop prediction models that are effective across a range of genetic material. To assess the potential for broadly focused GS training approaches, we investigated GS using a multi-population (MP) training set that combines data from smaller, random samples of five discrete breeding populations.

## Materials and methods

### Plant material and trial sites

A training set was composited from five perennial ryegrass breeding populations (designated Pop I to Pop V; 102–117 plants per population) from the Grasslands Innovation Ltd breeding programme, all of which were infected with the same fungal endophyte (*Epichloë festucae* var *lolii*) strain. Full detail of population development is provided in Supplementary material. Trials were sown with maternal HS seed harvested from 517 maternal parents. Six trials were sown at three New Zealand sites: Ruakura (Waikato region, northern New Zealand, 37.78°S, 175.32°E; Te Rapa peaty silt loam), Aorangi (Manawatu region, central New Zealand, 40.34°S, 175.46°E; Kairanga sandy loam) and Lincoln (Canterbury region, southern New Zealand, 43.38°S 172.62°E; Wakanui silt loam). Trials were sown in the Southern Hemisphere autumn or spring of 2013 (Supplementary Table S1). The unit for evaluation of HS families in the trials was a 1-m sown row of plants (0.2 g of seed per row; approximately equivalent to 14 kg ha^−1^ if a sward was sown at 7 rows per m), hereafter referred to as a plot. Plots were sown with 25–30 cm spacing between plots and 30 or 50 cm gaps at the end of the plots, depending on the trial. In the aligned breeding programme, HA measured in this type of plot was positively correlated (*r* = 0.77–0.83) with DMY in larger (1.5 × 5 m) plots (M. Z. Z. Jahufer, unpublished data).

Two trials were sown at each site. At both Ruakura and Aorangi, one trial (severe defoliation treatment; SEV) was grazed by sheep every 2 weeks through the summer period in 2014–2015 and 2015–2016. The other trial (standard treatment, STD) was also grazed but only when plants had reached the 2–3 leaf growth stage (see below). Mechanical mowing was used to defoliate under-grazed areas to a residual height of 5 cm, where needed. At the Lincoln site, one trial was STD and the other (irrigated treatment; IRR) was managed as STD except that it was watered by rainfall plus irrigation to approximately 40 mm of water weekly. All trials used a row–column experimental design with three replicates. Within each of the six trials, populations were blocked and families randomised in three replicates within these blocks. Repeated check lines were planted within and across replicate blocks. In all trials, soil fertility levels were adjusted to ensure nutrients were not limiting plant growth. Nitrogen was applied (15–30 kg N/ha) at each defoliation. Superphosphate fertilizer (8.8 kg P/ha) was applied in late autumn each year.

### Trait measurements

To assess DMY potential, HA was measured by cutting plots to a height of 5 cm. Harvested foliage was dried (80 °C for 48 h) and weighed to obtain HA as g DM per plot. Up to nine HA harvests were completed between summer 2014 and autumn 2016 in each trial (Supplementary Table S1). These were timed to occur at least once in summer, autumn and spring in a given year. However, not all seasons at all sites were sampled each year, due to sub-optimal growth conditions at certain times resulting in insufficient plant material for harvest (Supplementary Table S1). HA harvests were completed when plots were at the 2–3 leaf-growth stage, typically 3–4 weeks regrowth after the prior defoliation (dependent on season and site). The same defoliation management was used outside of when HA measurements were made.

Days-to-heading (DTH) was assessed at two sites (Ruakura and Lincoln) during spring 2015, using the Ruakura STD and Lincoln IRR trials. Both trials were closed to grazing from early September. Days-to-heading (DTH) was recorded as days from 1 November, with observations starting on that day and repeated every 2–3 days for the next 35 days. DTH was recorded when there were at least five heads in a plot that were fully emerged and these heads were appearing uniformly along the plot. Further detail of trial treatments are provided in Supplementary material.

### Statistical analysis of phenotypic data

Data were analysed using the linear mixed model option in GENSTAT (GenStat 2006). Analyses were conducted on the five individual populations and the full multi-population (MP) training set, for which data from all five populations were combined. HS families, replicates and row and column were treated as random effects. Years, harvests, sites and populations were treated as fixed effects. Treating the populations as fixed enabled assessment of differences between them for mean performance; within sites within treatments, within sites across treatments, across sites within treatments and across sites and treatments. Full details of the models are provided in Supplementary material.

Average HA, merging data from all individual harvests, was used for developing genomic prediction models. This was undertaken within- and across-sites and treatments, with ten HA traits produced: Rua STD (average HA in the Ruakura STD treatment); Rua SEV (average HA in the Ruakura SEV treatment); Aor STD (as per Rua STD, at the Aorangi site); Aor SEV (as per Rua SEV, at the Aorangi site); Lin STD (as per Rua STD, at the Lincoln site); Rua STD + SEV (average HA over both treatments at Ruakura); Aor STD + SEV (as per Rua STD + SEV, at Aorangi); Comb STD (average HA across all STD treatments); Comb SEV (average HA across both SEV treatments); and Comb STD + SEV (average HA over all sites and treatments). The Lin IRR trial showed no significant (*P* > 0.05) genetic variation for six of seven seasonal HA determinations completed (Supplementary Table S1), and so was not used. DTH data from the Ruakura and Lincoln sites were analysed as average performance across two sites, to generate an across-site assessment designated Comb DTH.

The adjusted HS family phenotypic means generated from each of the residual maximum likelihood (REML) analyses were based on best linear unbiased predictors (BLUP’s) (White and Hodge 1989). Estimation of the significance of the genetic variance component for each trait was based on the log-likelihood ratio test (Galwey 2006) and, where appropriate, the linear models also included family-by-year, family-by-harvest, family-by-site and family-by-treatment interaction effects. The row-column experimental design enabled adjustment for random error across replicates, columns and rows within replicates, and the repeated checks helped reduce trial spatial effects (Gleeson 1997; Gleeson and Kempton 1997).

For the MP analyses, variance components were used to estimate HS family mean repeatability (*R*), the upper limit of the degree of genetic determination (Falconer 1989). For individual population analyses, narrow sense heritability, *h*_n_^2^ was estimated on a family mean basis. The general equation used was:

1 $$R\;{\text{or}}\;h_{\text{n}}^{2} = \frac{{\sigma_{\text{g}}^{2} }}{{\sigma_{\text{g}}^{2} + \frac{{\sigma_{\varepsilon }^{2} }}{{n_{\text{r}} }}}},$$

where *σ*_g_^2^ is genotypic variation in the *R* estimation or ¼ additive variation (among HS family variation within individual populations) in the *h*_n_^2^ estimation; *σ*_*ε*_^2^, experimental error; *n*_r_, number of replicates. The denominator in Eq. 1 was expanded further with interaction component effects, depending on the analysis: *σ*_gy_^2^, genotype-by-year; *σ*_gh_^2^, genotype-by-harvest; *σ*_gt_^2^, genotype-by-treatment; *σ*_gI_^2^, genotype-by-site, and their associated divisors: *n*_y_, number of years; *n*_h_, number of harvests; *n*_t_, number of treatments; *n*_l_, number of sites.

Cluster analysis of the 517 HS families-by-five environment (Aor STD, Aor SEV, Rua STD, Rua SEV and Lin STD) BLUP matrix was carried out using a hierarchical agglomerative complete linkage procedure with squared Euclidean distance as a measure of dissimilarity (Wishart 1969). The Hartigan algorithm (Hartigan 1975) was used to determine the optimal number of clusters.

### DNA isolation and production of GBS libraries

DNA was isolated from approximately 100 mg of fresh leaf blade + pseudostem tissue for 577 training set mother plants, using a high-throughput method based on that described by Whitlock et al. (2008) with modifications including a final binding, washing and eluting DNA from AcroPrep™ Advance 96 Filter Plates (Pall Corporation, Ann Arbor, MI, USA). DNA quality was checked via visualisation on ethidium bromide stained 0.8% (wt/vol) agarose/TBE gels and then quantified using the Quant-iT™ PicoGreen^®^ dsDNA Assay Kit (Invitrogen, Carlsbad, CA). DNA concentrations were normalised to 20 ng/μl and subsequently used for GBS library preparation. GBS libraries were generated following the methodology of Elshire et al. (2011), with 100 ng of DNA digested using *Ape*KI (New England Biolabs, Ipswich, MA) and ligated to a unique barcoded adapter and a common adapter (99 ng). A total of six libraries were developed in 96-plex which included a blank and a common positive control sample. Each library was passed through a Pippin Prep™ DNA size selector (Sage Science, Beverly, MA, USA) to isolate fragments between 193 and 313 bp, which were then sequenced on two lanes of an Illumina HiSeq 2500 (Illumina, San Diego, CA, USA) at AgResearch Invermay, New Zealand.

### GBS data analysis

Raw reads from the 12 FASTQ data files were initially checked on the basis of read count statistics and then subjected to de-multiplexing, tag alignment and SNP calling based on the TASSEL 5.0 GBS pipeline (Glaubitz et al. 2014). SNP calling was conducted jointly for all six libraries, combining data for Pop I–V into a single analysis. A ryegrass reference genome was constructed by aligning a published ryegrass assembly (Byrne et al. 2015) onto the *Hordeum vulgare* genome (version 082214v1.27) to form ryegrass pseudochromosomes. Non-genic regions of the *H. vulgare* genome were masked prior to alignment to ensure alignment by gene synteny. Ryegrass contigs were aligned to the *H. vulgare* reference genome using Lastz version 7.0.1 (Harris 2007) from within Geneious 8 (http://www.geneious.com; Kearse et al. (2012) with parameters left at default. GBS tags were aligned to the constructed reference genome using Bowtie2 (Langmead and Salzberg 2012).

Duplicated samples, from the two lanes of data per library, were merged for genotyping calling, based on the binomial likelihood ratio method implemented in the TASSEL pipeline (Glaubitz et al. 2014) and 1,093,464 biallelic SNPs were retained after filtering using VCF tools (Danecek et al. 2011) with the criteria of 50% maximum missing data per site, minor allele frequency (MAF) > 0.05 and read depth > 1. Reference and alternative allele counts for the 1,093,464 SNPs were retrieved and exported for KGD analysis (Dodds et al. 2015). After filtering by Hardy–Weinberg disequilibrium (HWdiseq > − 0.05) 1,023,011 SNPs, with a mean read depth of 2.94, were obtained and used to compute a genomic relationship matrix (GRM) in that software, for GEBV estimation. HWdiseq filtering was used, as recommended by Dodds et al. (2015), as a tool to filter SNP potentially from duplicated or repetitive regions of the genome.

The retained 1,023,011 SNPs were also additionally filtered by allowable level of missing data per SNP locus, resulting in three datasets composed of different numbers of SNPs (Table 1). These SNP datasets were used for GEBV estimation using the RR, RF and GBLUP methods as described below. For all GEBV statistical methods except KGD, missing SNP genotypes were imputed using mean imputation (MNI) as explained below.

**Table 1:** SNP datasets produced for assessment of genomic selection statistical models

SNP set	Missing data per SNP site (%)	Total number of SNPs	Models tested
1	50	1,023,011	KGD, GBLUP, RF
2	10	249,546	GBLUP, RR, RF
3	1	43,966	GBLUP, RR, RF

*RR* ridge regression, *RF* random forest, *KGD* GBLUP using KGD-generated genomic relationship matrix

### Linkage disequilibrium (LD) analysis

LD was calculated by computing the pairwise LD measure $r^{2}$ (Hill and Robertson 1968) using a soon-to-be published methodology (Bilton et al. 2017), which accounts for uncertainty in the GBS genotypes associated with low-read depth (Bilton and Dodds 2016). SNPs were removed from the LD analysis for a given population if they had a minor allele frequency (MAF) < 0.05, 25% or more missing data, a mean read depth less than or equal to 20 or a Hardy–Weinberg disequilibrium estimate less than − 0.05 or less than − 0.8 times the squared MAF. Within each population, LD was calculated on all pairs of remaining SNPs which mapped to the same scaffold. The decay of $r^{2}$ with respect to distance was modelled using the equation,

2 $$E(r^{2} ) = \frac{1}{\alpha + 4\beta d} + \frac{1}{n},$$

where $n$ is the sample size, $d$ is the physical distance (in base pairs) between the SNPs and $\alpha$ and $\beta$ are parameters to be estimated (Weir and Hill 1980). As some genotypes are missing, the sample size $n$ was taken to be the number of individuals with no missing genotypes for a given pair of SNPs. Non-linear regression modelling was performed using the nls function in the statistical package R v3.3.0 (R Core Team 2017).

### Imputation of genotypic data

With the exception of KGD, the statistical methods below require a complete genotypic dataset. The initial GBS SNP marker data had up to 50% missing values per marker, therefore, we explored three methods, described in Rutkoski et al. (2013) for imputing missing data, mean imputation (MNI); expectation–maximisation algorithm (EMI); and random forest regression algorithm (RFI). We used packages impute, rrBLUP and MissForest of R software, respectively. When these imputation strategies were compared, with respect to GEBV predictive ability, on a sample of chosen traits and a set of SNP markers, all methods gave very similar results (data not presented). Therefore, we chose the simplest and most efficient approach, MNI, for further evaluations.

### Statistical methods for estimation of GEBVs

A number of statistical models have been developed for prediction of breeding or phenotypic values, and approaches can be collated into two categories, parametric and non-parametric (for a detailed review see de los Campos et al. (2013). We focused on simply modelling the BLUP-adjusted HS family means on marker information from the maternal parent for GEBV estimation. Assessment of statistical models was based on a final training set of *n* = 517 for which both phenotypic (HS family) and genotypic (maternal parent) data were available.

Suppose there are *n* individuals and m SNP markers. The first parametric model we used was ‘genomic BLUP’ (denoted here by GBLUP) and is defined as, *y* = *μ* + *Xu* + *e*, where y is the *n* × 1 vector of genotype BLUP’s (estimated in stage 1), *μ* is the *n* × 1 vector of grand mean, *X* is the *n* × *m* design matrix and *u* is the *m* × *1* vector of random marker effects with *u* ~ *N*(0, *σ*_*u*_^2^ *G*), where *G* is the *n* × *n* ‘genomic relationship matrix (GRM)’, and *e* is the *n* × 1 vector of random errors with *e* ~ *N*(0, *σ*_e_^2^ *I*), where *I* is the *n* × *n* identity matrix. With *M* = {*m*_*ij*_} representing the *n* × *m* marker matrix, *G* ∝ *MM* ^T^ and was calculated as *G* = *ZZ* ^T^/(2∑_*j*_ *p*_*j*_(1 − *p*_*j*_)), where *Z* is the (minor allele) adjusted SNP scores with elements (*m*_*ij*_ − 2p_j_), for *i*th individual (genotypes) and *j*th SNP, and *p*_*j*_ is the ‘minor allele frequency’ of the *j*th SNP. The GBLUP modelling was implemented using the ‘rrBLUP’ package (Endelman 2011) in R software (R Core Team 2017).

The second parametric model we considered, denoted by KGD, is a variation of GBLUP. Here, the GRM G was estimated using an approach proposed by Dodds et al. (2015). They provide a method which gives unbiased estimates of relatedness using SNPs assayed by GBS, which accounts for the depth (including zero depth) of the genotype calls.

We also utilised a linear regression model for estimating GEBV’s. With vectors and matrices as defined above, we may write this regression model as, *y* = *μ* + *Mu* + *e*. Since the number of predictors (SNP markers) exceeds the number of observations (genotypes), we needed to resort to ‘shrinkage’-based regression. A commonly used approach for genomic prediction is the ‘ridge regression’ model (denoted by RR), where a shrinkage parameter *λ* needs to be estimated. We used the package ‘glmnet’ (Friedman et al. 2010) of R software for implementing RR.

Finally, to explore a non-parametric and non-linear regression approach, we considered a machine-learning algorithm, ‘random forest’ regression (RF) (Breiman 2001), for GEBV estimation. We used 500 trees and 10% of the total no. of SNPs to find the best split at each node (the latter choice was made due the size of marker data used and the CPU time required for computations). The RF modelling was implemented using the package “ranger” of R software.

### Predictive ability via cross-validation

The predictive ability (PA) of the models (GBLUP, KGD-BLUP, RR and RF) was assessed via cross-validation. Here, the data were split into training and validation sets. We undertook two assessments of the MP-based models to estimate their PA: (1) within the breeding programme as a whole and (2) for each of the individual populations, Pop I–V.

For (1) we considered a tenfold cross-validation, where phenotypic (BLUP) data and corresponding genotypic (SNP marker based) data of the 517 individuals were randomly divided into ten subsets of similar size. Note here that the validation set was composed of individuals from all five populations. Models were fitted on the training set (i.e., nine subsets consisting of 90% of the data) and the GEBV’s predicted for the validation set (remaining subset). The PA of the models was computed as the Pearson’s correlation coefficient between predicted values (GEBV’s) and observed values (BLUP’s). For this set of cross-validations, bias of the different statistical methods for prediction was also assessed by measuring the slope of the regression of GEBV on BLUP.

For each trait and each SNP marker data, five replicates of the cross-validation scheme were considered (thus, resulting in five GEBV estimates for each genotype), enabling computation of empirical standard errors of PA and ensuring that results obtained were not due to random partitioning of data. Five replicates were deemed sufficient after it was determined that expanding to ten replicates made no difference to variability or PA (Supplementary Figures S5 and S6). To warrant precise comparisons, the four different statistical models were fitted in exactly the same cross-validation partitions. To compare similarities of these models across all chosen traits, a simple ANOVA with two factors (genomic prediction method and trait) was employed. The PA’s computed separately for each of the five tenfold cross-validation sets were considered as response.

**Fig. 1:**
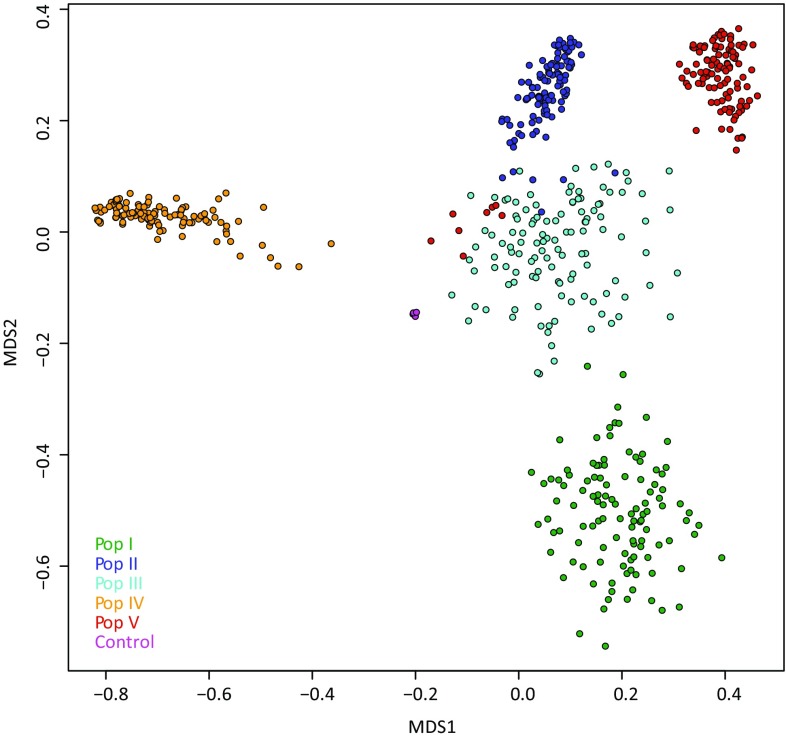
Multi-dimensional scaling ordination plot for a ryegrass training set made up of individuals (*n* = 566) from five breeding populations (Pop I–V). Six repeats of a control DNA sample (one per 96-plex GBS library created) are represented by purple dots near the centre of the image

For (2), estimating PA- of MP-based models within each of Pop I–V, we used a cross-validation scheme wherein the training set consisted of approximately 258 individuals balanced across all five individual populations, including about 50% of the individuals from the target (predicted) population. The validation set was composed of the remaining 50% of individuals from the target population. This approach ensured that the predicted population was represented equally in both the training and validation sets. The smaller size of the overall training set was necessary to ensure it was composed of a balanced number of individuals from all five individual populations. For each trait and each SNP marker data, 25 replicates of the cross-validation scheme were considered. Outlier individuals associated with Pop V (Fig. 1) were excluded.

### Simulation of genetic gain from GS

Simulations were implemented to compare genetic gain (∆G_c_) from one cycle of selection using conventional phenotypic HS family selection (HSF), against a GS strategy (A_p_WF_gs_-HS). The GS strategy simulated combined among-family selection based on phenotype and within-family selection using genomic prediction. The simulation used ‘real’ data generated from training population phenotyping trials, specifically: Rua STD for Pop II (*n* = 108 HS families), based on HA data from eight discrete harvests over a period of 27 months. This trait was chosen as it was based on the most complete set of seasonal data in the study (Supplementary Table S1). Pop II was selected as the best-performed population in this environment (Table 2).

**Table 2:** Genotypic (*σ*_g_^2^; for multi-population training set, MP) or additive (*σ*_a_^2^; for individual populations), genotype-by-harvest (*σ*_gh_^2^), genotype-by-year (*σ*_gy_^2^), genotype-by-treatment (*σ*_gt_^2^), genotype-by-site (*σ*_gl_^2^) and experimental error (*σ*_ε_^2^) variance components and their associated standard errors (± SE) and *R* (repeatability; for MP training set) or *h*_n_^2^ (narrow-sense heritability; for individual populations), estimated for HA (g DM per plot) and DTH (number of days after 25 October) traits

Trait	Training set	Mean	Range	*σ*_(g or a)_^2^	*σ*_gh_^2^	*σ*_gy_^2^	*σ*_gt_^2^	*σ*_gl_^2^	*σ*_ε_^2^	*R* or *h*_n_^2^
Rua	MP	25.2	13.5–36.8	13.6 ± 1.8	2.0 ± 0.7	19.7 ± 1.6	–	–	91.8 ± 1.5	0.47
STD	Pop I	21.2	11.8–30.2	15.7 ± 3.72	ns	10.7 ± 2.67	–	–	57.1 ± 2.93	0.65
	Pop II	32.2	22.9–39.9	10.3 ± 2.41	ns	8.0 ± 1.95	–	–	66.1 ± 2.58	0.67
	Pop III	25.3	15.4–36.5	15.1 ± 3.07	ns	11.7 ± 2.25	–	–	77.6 ± 2.85	0.60
	Pop IV	22.1	14.9–30.3	12.5 ± 3.60	ns	10.1 ± 3.45	–	–	55.9 ± 4.82	0.61
	Pop V	24.9	13.9–37.9	26.7 ± 4.95	ns	11.5 ± 2.50	–	–	73.0 ± 3.17	0.73
Rua	MP	29.6	17.3–48.9	30.3 ± 3.2	ns	12.0 ± 1.9	–	–	101.1 ± 2.1	0.67
SEV	Pop I	26.7	15.4–41.1	41.4 ± 8.14	ns	ns	–	–	94.2 ± 7.29	0.84
	Pop II	39.8	32.0–52.4	21.9 ± 4.10	ns	ns	–	–	76.8 ± 4.33	0.77
	Pop III	30.6	20.2–41.7	30.1 ± 4.94	ns	ns	–	–	92.6 ± 4.95	0.80
	Pop IV	26.1	16.0–47.0	42.7 ± 10	ns	ns	–	–	60.6 ± 6.71	0.89
	Pop V	26.1	16.1–37.9	32.5 ± 5.93	ns	6.6 ± 3.03	–	–	84.1 ± 5.13	0.76
Aor	MP	58.9	53.0–63.4	4.6 ± 2.2	ns	11.1 ± 2.8	–	–	172.4 ± 3.5	0.23
STD	Pop I	52.2	44.6–58.7	10.9 ± 4.28	ns	8.9 ± 4.14	–	–	80.5 ± 5.23	0.55
	Pop II	52.4	48.0–57.1	5.5 ± 2.05	ns	ns	–	–	85.7 ± 4.24	0.54
	Pop III	51.4	44.6–60.6	8.6 ± 3.19	ns	12.7 ± 3.76	–	–	97.6 ± 4.40	0.42
	Pop IV	54.4	49.5–59.2	8.1 ± 3.19	ns	ns	–	–	103.4 ± 9.50	0.59
	Pop V	57.1	49.0–64.4	8.0 ± 3.6	10.0 ± 4.70	11.9 ± 5.00	–	–	117.3 ± 6.50	0.34
Aor	MP	38.4	24.0–52.1	28.7 ± 3.0	10.2 ± 2.2	ns	–	–	128.3 ± 2.8	0.73
SEV	Pop I	38.2	17.4–53.5	63.8 ± 10.25	22.1 ± 5.03	ns	–	–	75.9 ± 4.82	0.85
	Pop II	41.9	30.6–54.3	35.2 ± 5.60	11.6 ± 5.10	ns	–	–	101.0 ± 5.20	0.79
	Pop III	39.9	23.6–55.4	39.0 ± 5.73	14.8 ± 3.71	ns	–	–	83.0 ± 4.08	0.80
	Pop IV	39.6	27.5–49.0	25.4 ± 5.26	ns	ns	–	–	57.6 ± 5.09	0.89
	Pop V	32.5	21.7–46.8	45.4 ± 7.24	28.2 ± 5.18	ns	–	–	85.2 ± 4.59	0.76
Lin	MP	43.7	35.7–52.2	10.5 ± 2.0	ns	11.4 ± 2.4	–	–	124.3 ± 3.0	0.45
STD	Pop I	46.4	39.7–51.8	11.6 ± 4.43	ns	12.0 ± 5.33	–	–	83.1 ± 6.77	0.52
	Pop II	40.8	33.6–45.8	12.2 ± 2.95	ns	ns	–	–	69.3 ± 4.17	0.76
	Pop III	43.8	35.8–52.7	9.9 ± 3.05	ns	5.8 ± 2.88	–	–	71.5 ± 3.95	0.59
	Pop IV	–	–	ns	ns	ns	–	–	63.2 ± 11.19	–
	Pop V	48.1	42.6–54.1	12.8 ± 3.68	ns	8.3 ± 4.05	–	–	78.1 ± 5.10	0.60
Rua	MP	28.2	16.2–42.5	14.3 ± 2.0	ns	11.6 ± 1.1	11.7 ± 1.3	–	98.3 ± 2.2	0.50
STD + SEV	Pop I	23.5	12.7–33.4	12.5 ± 4.20	ns	ns	16.0 ± 3.80	–	81.7 ± 3.20	0.54
	Pop II	–	–	ns	ns	2.8 ± 1.21	18.6 ± 3.63	–	82.0 ± 2.50	–
	Pop III	28.1	18.1–40.3	16.3 ± 3.22	ns	4.3 ± 1.40	5.7 ± 1.74	–	96.1 ± 2.73	0.68
	Pop IV	–	–	ns	ns	2.9 ± 1.91	37.5 ± 8.02	–	71.9 ± 3.41	–
	Pop V	26.8	16.6–38.9	17.0 ± 4.93	ns	4.9 ± 1.64	19.6 ± 3.94	–	92.9 ± 3.03	0.53
Comb	MP	–	–	ns	3.1 ± 0.6	11.4 ± 1.0	–	22.7 ± 1.6	119.8 ± 1.3	–
STD	Pop I	35.3	27.9–42.2	4.9 ± 2.19	ns	4.1 ± 1.46	–	11.2 ± 2.28	77.9 ± 2.79	0.40
	Pop II	37.0	32.6–42.5	2.9 ± 1.35	ns	2.9 ± 0.97	–	7.6 ± 1.46	73.4 ± 2.02	0.35
	Pop III	37.8	32.7–43.1	4.2 ± 1.61	ns	ns	–	9.3 ± 2.01	167.6 ± 3.82	0.40
	Pop IV	–	–	ns	ns	4.5 ± 2.18	–	12.0 ± 3.15	75.1 ± 4.11	–
	Pop V	38.5	31.7–46.8	8.6 ± 2.65	ns	5.1 ± 1.42	–	10.7 ± 2.02	84.6 ± 2.50	0.53
Comb	MP	33.0	21.0–45.4	13.5 ± 2.4	2.6 ± 0.8	2.7 ± 1.0	–	20.0 ± 2.1	117.3 ± 1.72	0.47
SEV	Pop I	32.4	18.2–43.6	29.1 ± 7.62	ns	ns	–	24.2 ± 5.93	107.2 ± 5.14	0.66
	Pop II	38.5	30.5–47.9	10.8 ± 3.44	ns	ns	–	12.7 ± 3.44	126.2 ± 3.42	0.52
	Pop III	33.3	24.6–41.3	33.3 ± 4.31	ns	ns	–	14.7 ± 3.63	133.0 ± 4.53	0.75
	Pop IV	33.0	26.7–41.5	14.1 ± 5.22	ns	ns	–	7.6 ± 3.20	132.8 ± 7.70	0.65
	Pop V	28.5	19.0–40.0	20.0 ± 4.6	ns	ns	–	11.1 ± 3.12	100.4 ± 3.82	0.71
Comb	MP	–	–	ns	0.8 ± 0.3	5.5 ± 0.6	11.5 ± 1.0	19.9 ± 1.3	124.4 ± 1.1	–
STD + SEV	Pop I	35.8	24.9–44.6	7.6 ± 3.32	ns	2.0 ± 0.93	14.8 ± 2.8	11.9 ± 2.25	79.4 ± 2.30	0.37
	Pop II									
	Pop III	35.7	29.4–41.6	4.8 ± 1.78	ns	2.0 ± 0.70	4.9 ± 1.21	9.4 ± 1.46	89.6 ± 1.80	0.39
	Pop IV	–	–	ns	ns	2.3 ± 1.31	8.2 ± 2.30	8.4 ± 2.25	71.6 ± 3.05	–
	Pop V	36.6	28.9–45.0	10.0 ± 2.73	ns	2.5 ± 0.86	6.3 ± 1.48	8.5 ± 1.58	85.8 ± 2.00	0.55
Comb	MP	16.2	11.1–21.2	6.1 ± 0.8	–	–	–	0.2 ± 0.02	18.5 ± 0.7	0.66
DTH	Pop I	–	–	ns	–	–	–	5.2 ± 1.97	12.8 ± 1.59	–
	Pop II	17.0	10.6–22.0	6.0 ± 1.26	–	–	–	1.7 ± 0.78	8.3 ± 0.77	0.73
	Pop III	16.7	12.5–21.6	4.4 ± 1.27	–	–	–	ns	17.1 ± 1.34	0.61
	Pop IV	16.7	6.9–17.3	13.3 ± 3.71	–	–	–	ns	21.6 ± 4.08	0.79
	Pop V	24.2	21.1–26.6	2.3 ± 1.12	–	–	–	ns	17.3 ± 1.71	0.44

ns indicates a variance component was recorded but was not statistically significant (*P* > 0.05). Results are given for analysis of populations as a MP training set and as individual populations (Pop I–V). Where there was no significant (*P* > 0.05) *σ*_(g or a_)^2^ for a training set, results are not shown (including all training sets for the trait Aor STD + SEV)

*STD* standard grazing management, *SEV* severe summer grazing management, *Comb* data combined across locations and/or treatments

For simulating A_p_WF_gs_-HS, a range of genomic PAs were assumed (*r* = 0.10–0.50, in steps of 0.10) in addition to the PA of 0.27 estimated by cross-validation for Rua STD in Pop II (Fig. 4). Among-family selection intensity (*k*_f_) was fixed at 1.40 (top-ranked 20% of families) for HSF and all A_p_WF_gs_-HS simulations, while three different within-family selection intensities were tested at each PA for A_p_WF_gs_-HS: *k*_w_ = 2.67 (top-ranked 1% of individuals within family), *k*_w_ = 2.27 (3% of individuals) and *k*_w_ = 2.06 (5% of individuals). The estimation of genetic gain using within-family genomic selection was based on a modification of the equation proposed by Casler and Brummer (2008), for among-and-within family selection, where within-family selection is based on a secondary trait, X, the primary trait being Y. The equation used in DeltaGen:

3 $${\text{A}}_{\text{p}} {\text{WF}}_{\text{gsy-HS}} = k_{\text{f}} c_{\text{f}} \frac{{\frac{1}{4}\sigma_{\text{AY}}^{2} }}{{\sigma_{\text{PF}} }} + k_{\text{w}} c_{\text{w}} h_{\text{X}} r_{\text{A-XY}} \frac{\surd 3}{2}\sigma_{\text{AY}} ,$$

where ${\text{A}}_{\text{p}} {\text{WF}}_{\text{gsy-HS}}$ is the predicted ∆*G* using a combination of phenotypic among-HS family selection and within-HS family GS; *σ*_AY_^2^, additive genetic variance for the trait Y under selection; *σ*_AY_, standard deviation of additive genetic variance for trait Y; *σ*_PF_, among-family phenotypic standard deviation for trait Y; *k*_f_ and *k*_W_, among- and within-HS family selection intensity, respectively; *c*_f_ and *c*_W_, among- and within-HS family parental controls, respectively [for HS family selection *c* = 0.5; Casler and Brummer (2008)]; *h*_X_, square root of heritability for secondary trait X and $h_{\text{X}}$ is assumed to be 1; *r*_A-XY_, genomic PA.

Expected ∆*G* for HSF selection was estimated using the equation proposed by Casler and Brummer (2008) for forage crops:

4 $$\Delta G = k_{\text{f}} c\frac{{\frac{1}{4}\sigma_{\text{AY}}^{2} }}{{\sigma_{\text{PF}} }},$$

where *σ*_AY_^2^ is additive genetic variance and *c* = 0.5.

## Results

### GBS data and population structure

SNP data were acquired via GBS for 566 of the 577 mother plants submitted for analysis. Lane-to-lane sequence variation of the read counts was minimal, with the coefficient of variation of the barcoded read counts in each lane between 12 and 20%, confirmed also by the positive control sample located on each plate (see below). SNP calling was conducted jointly for all populations, Pop I–V. Initial filtering yielded 1,093,464 SNPs across all populations and this was reduced to 1,023,011 following an additional filter for Hardy–Weinberg disequilibrium (HWdiseq > − 0.05). The mean missing rate per SNP of this subset was 0.24 and the mean read depth 2.94. The overall MAF distribution of the selected SNPs is shown in Supplementary Figure S2.

A genomic relationship matrix (*S*) of dimension 566 × 566 was derived using the KGD analysis package, based on the set of 1,023,011 SNPs. Multidimensional scaling (MDS) was applied to its distance matrix (1 − *S*), revealing the structure amongst the five individual populations of the MP training set (Fig. 1). With few exceptions, genotypes clustered by population and populations were grouped discretely. The relative positions of the five populations reflected their known breeding histories (Dr. Alan Stewart, pers comm). Eight outlying individuals associated with Pop V and six with Pop II were confirmed via repeat genotyping not to have been mis-labelled (data not presented). The first MDS ordinate separated Pop IV, a population characterised by early DTH, from the other four individual populations which all had later DTH (Table 2). The second ordinate further separated Pops I, II, III and V, with a closer relationship indicated amongst Pops II, III and V. Positive control samples of a single genotype, repeated in all six GBS libraries and run in different sequencing lanes, landed at the same position in space on the MDS plot, indicating consistency of GBS and SNP-calling across the six libraries. The genomic relationship matrix, *S*, was subsequently used for genomic prediction modelling using the KGD model. Additional filtering on missing data per SNP site was applied (Table 1), resulting in three SNP datasets with SNP numbers ranging from ca. 44 K to 1.02 M.

**Fig. 2:**
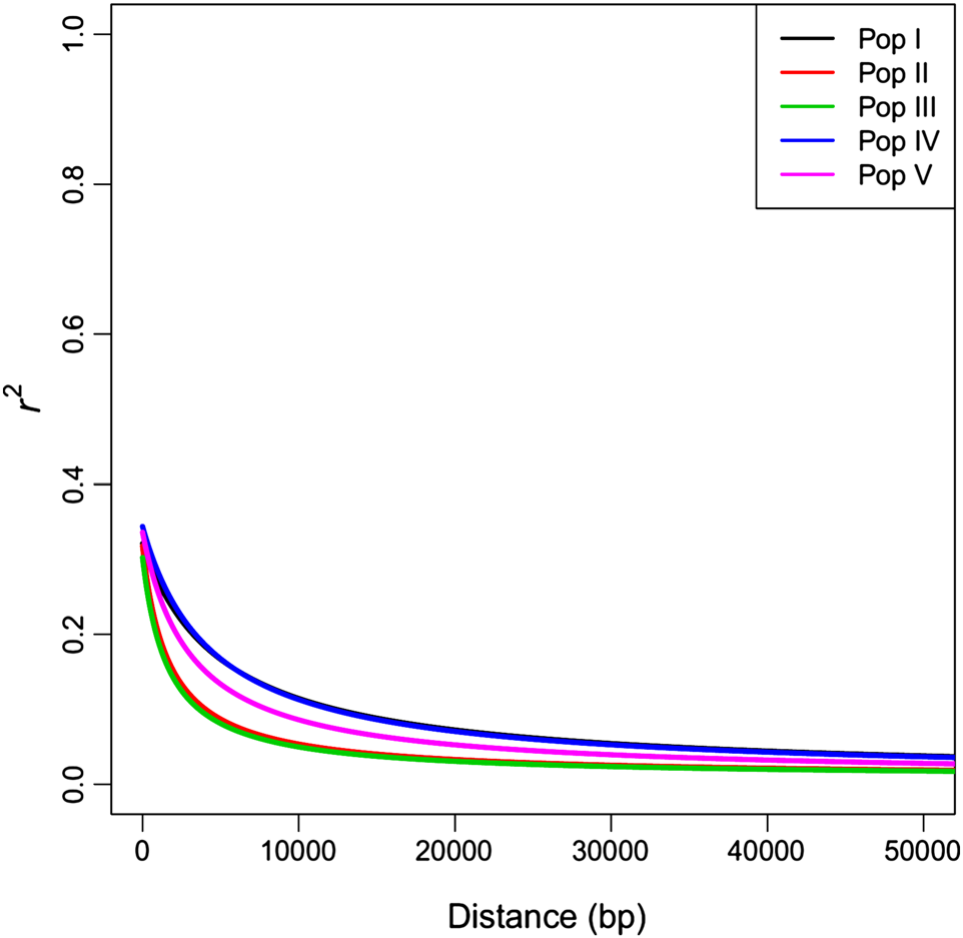
Decline in linkage disequilibrium, measured as *r* ^*2*^ against distance in base pairs (bp), for the five populations making up the multi-population (MP) perennial ryegrass training set. The lines are non-linear regression models estimated for each of Pop I–V. These are based on pairwise *r* ^*2*^ values between all SNPs mapped to the same scaffold, for all chromosomes within the population (Supplementary Figure S3)

A summary of results from linkage disequilibrium (LD) analysis of the five individual populations in the MP training set is shown in Fig. 2. LD decay in all populations was rapid, decaying to below *r* ^*2*^ of 0.25 after 366–1750 base pairs (bp). The rates of decay varied amongst the populations. Pop III was characterised by the shortest extent of LD (*r* ^*2*^ = 0.25 at 366 bp), followed by Pop II (477 bp) and the remaining populations had longer LD—Pop I (1506 bp) Pop IV (1750 bp) and Pop V (1100 bp). These observations align with what is understood about the breeding history of two of the contrasting populations. Pop I was based on only six pair crosses between randomly selected individuals from two cultivars, whereas Pop III originated with contributions from a larger number and greater diversity of parents—a polycross of 80 randomly selected individuals from six different cultivars. After advancement to F_2_, both underwent multiple cycles of selection prior to their inclusion in the MP training set.

### Phenotypic data

Data from 28 seasonal HA harvests (Supplementary Table S1) were used to derive estimates of average HA within each site and treatment combination, and across site and treatments. Nine discrete HA traits were generated and variance components (Table 2) and BLUP’s estimated. These BLUP’s represented the estimated breeding value for the mother plants polycrossed, within each of the five populations, to generate the HS families.

For the MP training set (*n* = 517 HS families) significant (*P* < 0.05) *σ*_g_^2^ amongst families was indicated for seven of the HA traits as well as DTH estimated across two environments (Comb DTH) (Table 2). There was no significant (*P* > 0.05) *σ*_g_^2^ for HA traits Comb STD (combined data from all standard grazing sites) or Comb STD + SEV (combined data from all grazing treatments and sites). Significant genotype-by-location (*σ*_gl_^2^) and genotype-by-treatment (*σ*_gt_^2^) interactions (Table 2) indicated variation in the relative ranking amongst HS families due to trial location (HA traits, Comb DTH) or summer grazing management (HA traits). HS family mean *R*, estimating the upper limit of heritability, was high for Comb DTH as well as for HA under severe grazing treatment (compared with standard grazing) and the same trend was observed for *h*_n_^2^ in individual populations. Further description of variance components for both the MP training set and individual populations, including genotype-by-environment interactions, as well as *R* and *h*_n_^2^ values, are provided in Supplementary material.

### Genomic prediction using GBS SNP data

PA of genomic prediction models was assessed by cross-validation at two levels. First, models based on the MP training set were assessed for prediction of individuals sampled from the MP without consideration of individual population (prediction at the breeding programme level). For KGD, GBLUP and RF a full set of 1,023,011 SNPs were used in the models. Due to computational limitations, only a smaller set of 249,546 SNPs was able to be used for RR.

**Fig. 3:**
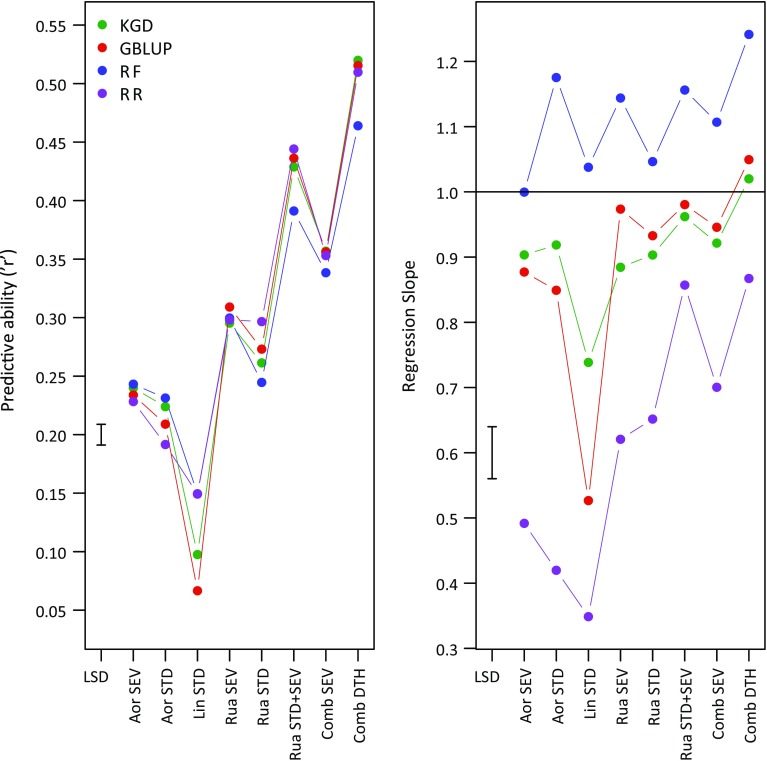
Mean (*n* = 5) tenfold cross-validation predictive ability in a multi-population training set (MP) for seven HA traits and DTH, determined using four statistical models and assessed as **a** Pearson’s correlation between observed phenotype (BLUP) and GEBV; **b** slope of the regression of GEBVs on BLUPs. *RR* ridge regression, *RF* random forest, *KGD* GBLUP using KGD genomic relationship matrix. RR models used 249,546 SNPs (largest SNP dataset able to be dealt with computationally by this method), while GBLUP, KGD and RF used 1,023,011 SNPs. Differences between any two statistical methods for each trait > LSD bar are significant at *P* < 0.05. In **b** statistical methods with a slope of regression of GEBVs on BLUP-adjusted means ≈ 1 are regarded as providing unbiased estimates of BLUPs. Lines are used on the plots not to infer continuity between the points but to clearly illustrate differences amongst the statistical methods

The highest PA amongst traits, determined as the Pearson’s correlation between observed phenotype (BLUP) and GEBV, was for Comb DTH (Fig. 3). Across all four statistical methods, mean *r* for Comb DTH was 0.50. Amongst HA traits, there was substantial variation in PA, ranging from Lin STD (mean *r* = 0.12 over the four methods), Aor STD (0.21), Aor SEV (0.24), Rua STD (0.27) up to Rua SEV (0.30). Combining data from certain sites and/or treatments generated higher PA: amalgamation of standard and severe treatment HA data from the Ruakura trials (Rua STD + SEV) had a mean PA of *r* = 0.43 and combining data from the severe grazing treatments at Aorangi and Ruakura (Comb SEV) resulted in a mean PA of *r* = 0.36.

Amongst HA traits and Comb DTH there were small (Fig. 3) but significant (Table 3) differences in PA when comparing the four statistical methods. RF overall ranked lowest in PA for five of eight traits, most significantly Comb DTH and Rua STD + SEV. The two GBLUP methods were statistically inseparable, except for Lin STD, and were significantly outperformed by another method for only two traits (Lin STD and Rua STD). The slope of the regression of GEBV on BLUP was determined to assess the bias of a statistical method, with unbiased models expected to have a slope coefficient of 1. The RF method over-estimated BLUP’s for all traits except for Aor SEV (Fig. 3), while all other methods tended to under-estimate, particularly RR. KGD and GBLUP methods for traits Rua SEV, Rua STD + SEV, Comb SEV and Comb DTH came close to being unbiased. In this dataset, there was also no obvious relationship between PA and *R*.

**Table 3:** Analysis of variance results considering two measures of genomic predictive ability, the Pearson’s correlation between BLUP’s and GEBV’s and the slope of the regression of GEBV’s on BLUP’s, with statistical method and trait as factors

Accuracy measure	Source	*df*	SS	MS	*F* value	*P* value
Pearson correlation	Method	3	0.0039	0.0013	6.50	0.0004
	Trait	7	2.1105	0.3015	1505.95	< 0.0001
	Method × trait	21	0.0535	0.0026	12.72	< 0.0001
	Residual	128	0.0256	0.0002		
Regression slope	Method	3	4.9230	1.6409	404.69	< 0.0001
	Trait	7	1.8710	0.2673	65.92	< 0.0001
	Method × trait	21	0.7570	0.0360	8.89	< 0.0001
	Residual	128	0.5190	0.0041		

**Fig. 4:**
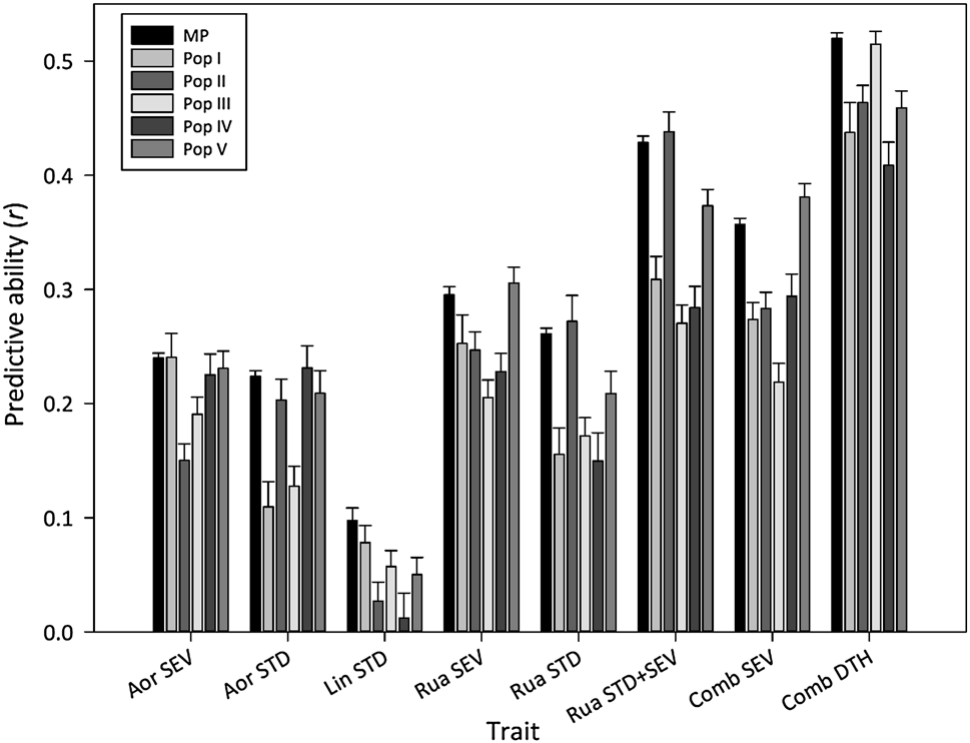
Cross-validation predictive ability (mean of *n* = 25 twofold cross-validations) in individual populations (Pop I–V) for seven HA traits and DTH. For each population, predictive ability is based on genomic prediction models developed from a multi-population (MP) training set, using the KGD statistical method. Prediction accuracies by KGD in the full MP training set (Fig. 3), are represented here (black symbol) for comparison. Error bars are SE

MP genomic prediction models were also assessed by cross-validation for PA within the respective individual populations, Pop I–V, using KGD. This reflects the situation in which a breeder applies a genomic prediction model developed with the MP training set, into one specific breeding population. PA varied amongst the five populations (Fig. 4) but the direction of *r* in the individual populations was consistent with that observed in the full MP training set (black symbols in Fig. 4) and the magnitude was similar albeit reduced by 10–32% (depending on population and trait): very low to low PA for within-environment HA traits, Lin STD (*r* = 0.01–0.08, mean 0.04), Aor STD (0.11–0.23, mean 0.18), Aor SEV (0.15–0.24, mean 0.21), Rua STD (0.21–0.31, mean 0.19) and Rua SEV (0.21–0.31, mean 0.25); and higher for Comb DTH (0.41–0.51, mean 0.46) and HA traits where data were merged from more than one environment, Rua STD + SEV (0.27–0.44, mean 0.34) and Comb SEV (0.22–0.38, mean 0.29). No one of the five populations ranked consistently highest for PA across all traits (Fig. 4) but Pop V was highest on average, followed by Pop II, Pop I, and Pops III and IV ranked last equal. Similar PA values were observed using three other statistical modelling approaches, GBLUP, RR and RF (data not presented).

The influence of SNP density on PA was investigated to a limited extent. SNP datasets of different sizes were generated (Table 1) but this was achieved by manipulating the allowable missing data level so that SNP datasets of different sizes also potentially varied in terms of the reliability of SNP genotype calls. For all statistical methods there was minimal effect of SNP number for predicting traits in the MP training set (Table 4). PA’s estimated with the smallest SNP dataset (44 K) were not significantly different (*P* < 0.05) to those estimated using the largest SNP dataset (1.02 M). A comprehensive summary of the effects of missing SNP data rate and read depth, by statistical method, is provided in Supplementary Table S3.

**Table 4:** Mean (*n* = 10) cross-validation predictive ability in a multi-population (MP) training set using three different SNP sets for seven HA traits and Comb DTH

Trait	RF			GBLUP			RR		KGD
	Set 1	Set 2	Set 3	Set 1	Set 2	Set 3	Set 1	Set 2	Set 3
Aor STD	0.23 (0.007)	0.23 (0.008)	0.23 (0.003)	0.23 (0.006)	0.22 (0.006)	0.21 (0.006)	0.22 (0.009)	0.19 (0.007)	0.22 (0.005)
Aor SEV	0.26 (0.004)	0.26 (0.004)	0.24 (0.005)	0.24 (0.004)	0.24 (0.005)	0.23 (0.006)	0.21 (0.005)	0.23 (0.005)	0.24 (0.004)
Lin STD	0.11 (0.006)	0.14 (0.005)	0.15 (0.010)	0.10 (0.003)	0.10 (0.003)	0.07 (0.009)	0.13 (0.006)	0.15 (0.006)	0.10 (0.011)
Rua STD	0.26 (0.007)	0.25 (0.007)	0.25 (0.004)	0.26 (0.005)	0.27 (0.006)	0.26 (0.005)	0.28 (0.006)	0.30 (0.007)	0.26 (0.005)
Rua SEV	0.32 (0.008)	0.31 (0.006)	0.30 (0.006)	0.31 (0.006)	0.31 (0.005)	0.31 (0.005)	0.28 (0.010)	0.30 (0.008)	0.30 (0.007)
Rua STD + SEV	0.40 (0.006)	0.40 (0.005)	0.39 (0.004)	0.43 (0.006)	0.44 (0.007)	0.44 (0.007)	0.44 (0.007)	0.44 (0.008)	0.43 (0.006)
Comb SEV	0.36 (0.007)	0.35 (0.006)	0.34 (0.005)	0.35 (0.007)	0.36 (0.007)	0.36 (0.007)	0.33 (0.011)	0.35 (0.010)	0.36 (0.006)
Comb DTH	0.47 (0.004)	0.47 (0.004)	0.46 (0.005)	0.52 (0.002)	0.52 (0.003)	0.52 (0.003)	0.51 (0.002)	0.51 (0.003)	0.52 (0.005)

Set 1 = 43,966 SNPs (1% missing data per SNP site). Set 2 = 249,546 SNPs (10% missing data per SNP site). Set 3 = 1,023,011 SNPs (50% missing data per SNP site)

*RR* ridge regression, *RF* random forest, *KGD* GBLUP using KGD genomic relationship matrix

### Simulation of genetic gain under a GS breeding strategy

**Fig. 5:**
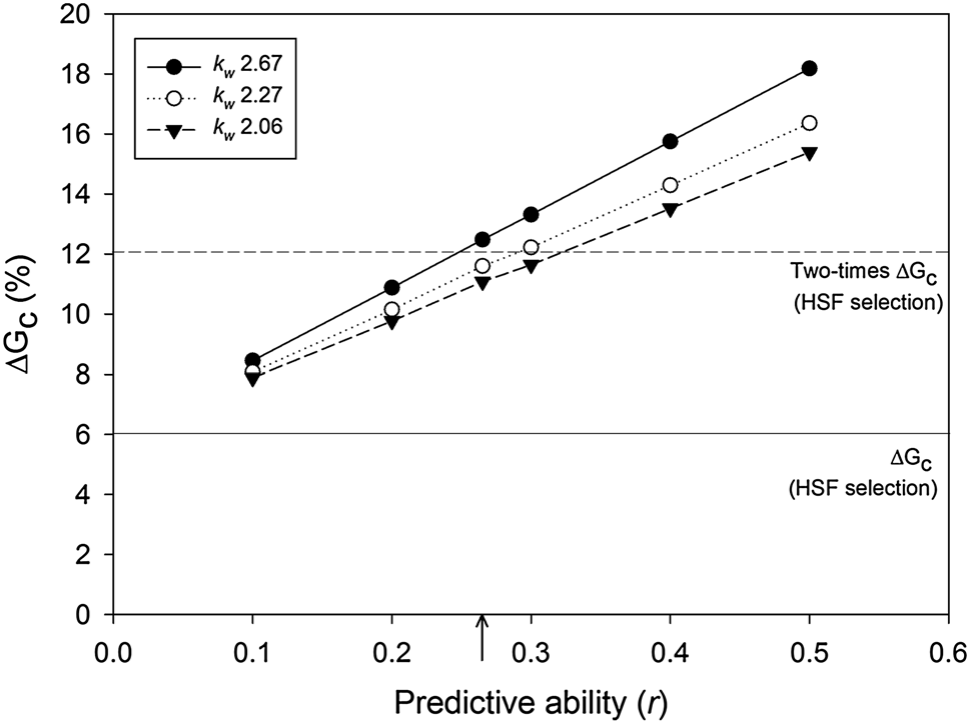
Predicted rates of genetic gain (∆*G*_c_) for HA based on data from the evaluation of 108 half-sibling (HS) families of perennial ryegrass Pop II in the Rua STD environment. HS family selection (HSF) is compared with among-HS family phenotypic selection and within-family genomic selection (A_P_WF_gs_-HS). ∆*G*_c_ is estimated at six levels of genomic-predictive ability (*r*), including the value estimated by cross-validation for Rua STD HA in Pop II (0.27, indicated by arrow). Three within-HS family selection intensities (*k*_w_ = 2.06, 2.27 and 2.67, equivalent to selecting the top 5, 3 and 1% of individuals, respectively) are tested at each *r*. Among-HS family selection intensity is fixed at *k*_f_ = 1.40 (equivalent to selecting top 20% of HS families) for all HSF and A_P_WF_gs_-HS scenarios. The solid horizontal line indicates ∆*G*_c_ for HSF selection (6.02) and the dotted line shows two times that rate (12.04)

Figure 5 illustrates the modelled impact on ∆*G*_c_ when using a genomic prediction model to select the top-ranked individuals by GEBV within the best 20% of HS families (A_p_WF_gs_-HS), as compared to randomly selecting individuals within those families (HSF). HSF in Pop II achieved ∆*G*_c_ of 6.02% for the Rua STD HA trait, with a single selection cycle. Applying genomic prediction to select the top individuals within the best 20% HS families resulted in ∆*G*_c_ increasing from 6.02% to a minimum of 7.89%, assuming the lowest genomic PA (0.10) and lowest selection intensity (equivalent to selecting the top 5% of individuals within-family). This increased to a maximum ∆*G*_c_ of 18.18% when the highest PA (0.50) and selection intensity (picking top 1% of individuals within family) combination was applied. This range is equivalent to a 31–202% improvement in ∆*G*_c_ by applying A_p_WF_gs_-HS over HSF. The effect of using a higher selection intensity was amplified as PA increased, as seen by the relative slopes of the three selection intensity lines (Fig. 5). At PA of 0.10, moving from picking the top 5% of individuals within-family to the top 1%, increased ∆*G*_c_ by 0.56%. At PA of 0.50 the same change in selection intensity increased ∆*G*_c_ by 2.78%. Taking the PA of 0.27, as estimated by cross-validation for Rua STD in Pop II (Fig. 4), and applying the highest within-family selection intensity in the A_p_WF_gs_-HS scenario, ∆*G*_c_ increased by twofold over HSF (Fig. 5).

## Discussion

We describe an empirical assessment of GS for perennial ryegrass using GBS SNP data in conjunction with a training set composited from multiple breeding populations, with the principal focus on HA as a measure of DMY potential. The GS training experiment emulates a genotypic recurrent selection breeding scheme in which the genetic merit of an individual is estimated by assessing the average performance of maternal HS progeny in sown field plots, for multiple HA harvests (Conaghan and Casler 2011) in multiple environments.

A multi-environment phenotypic dataset was acquired that reflects grass production systems in New Zealand, wherein pastures are typically grazed for most seasons of the year, there is substantial regional variation in terms of climate, soil characteristics, insect and disease pressures (Chapman et al. 2017) and perennial ryegrass-based pastures are expected to be productive for multiple years (Tozer et al. 2011). Overall, the phenotyping experiment provided an effective basis for the development of genomic prediction models. Significant genetic variation was detected for HA measured in plots amongst HS families within five trial environments (defined by location and grazing treatment), with moderate to high *R*. Particularly, high *R* (0.67–0.73) occurred in environments where frequent summer defoliation was applied. Severe grazing during summer, through the imposition of short grazing intervals and/or low grazing height, can lead to loss of plant vigour in ryegrass pastures (Cosgrove 2011; Brougham 1960) and may negatively influence the persistence of dry matter production in subsequent seasons (Brougham 1970). Our findings indicate a strong genetic component for HA, particularly where plants have been previously stressed by frequent summer defoliation, and therefore, potential for genetic improvement of ryegrass yield and persistence under sub-optimal conditions.

In general, genomic selection PA increases with training set size (Asoro et al. 2011; Cericola et al. 2017; Hayes et al. 2009b; VanRaden et al. 2009) and is affected by the magnitude of genetic relatedness between training and selection or validation populations (de Roos et al. 2009; Habier et al. 2007; Taylor 2014). In forage species, DMY is only realistically phenotyped in sown plots and in this regard training set size represents a practical constraint because limited numbers (e.g., often < 100) of such records from half- or full-sibling families are typically available for a given population in one generation. Moreover, breeding programmes can consist of several breeding populations, with varying levels of genetic relatedness, and it would be efficient to have genomic prediction models that are applicable to the full extent of genetic material in the programme. Combining data from multiple populations is a potential strategy for increasing training set size as well as ensuring representation of the full genetic base of the breeding programme in the training set. A combined population training set may also better support GEBV estimation for populations not represented in the training set (Pryce et al. 2011). In the current study, genomic prediction models constructed with a moderately sized MP training set were predictive of HA traits, and DTH, to varying levels of accuracy, when assessed at either the breeding programme level (treating the MP training set as a single-target population) or in each of the individual populations that made up the MP training set. PA in the full MP set for within-environment HA traits ranged from *r* = 0.07–0.31. This corresponds with PA reported for HS family-derived DMY datasets in other studies, including perennial ryegrass (Grinberg et al. 2016) (*r* = 0.01–0.32), alfalfa (Annicchiarico et al. 2015) (*r* = 0.0–0.35) and switchgrass (Ramstein et al. 2016) (*r* = 0.09–0.50). High PA (up to *r* = 0.58) for Comb DTH, almost twice that of within-environment HA traits, is in agreement with studies in other monocot plant species (Spindel et al. 2015; Thavamanikumar et al. 2015; Zhao et al. 2014) and is likely influenced by simpler genetic architecture and higher heritability (Fè et al. 2015a; Elgersma 1990).

MP-based models were also effective within each of the individual populations in the training set, although PA was variable amongst populations and on average lower (10–32%) than PA observed in the training set as a whole. Given the small size of the individual populations, it is difficult to compare MP-based prediction models with within-population models, as cross-validation correlations in the latter could only be based on very small validation sets; when we investigated this we observed nearly half within-population models yielded *r* ≤ 0.05 and high standard error for those estimates (data not presented). In dairy cattle, where robust comparison of pure-against multi-breed approaches has been completed, the experience from various studies is of either a small increase or no change in PA when using a multi-breed training set for predicting GEBVs within a single breed. The efficacy of a multi-breed training approach was influenced by trait architecture and the statistical model used (Erbe et al. 2012; Hayes et al. 2009a; Pryce et al. 2011) and was most advantageous for breeds with few individuals represented in the training set (Olson et al. 2012). In plant species, training sets for genomic prediction are often smaller than for animals and, in empirical studies where training set composition has been manipulated to achieve higher numbers, the effect of size on PA has not been straightforward. For example, Grinberg et al. (2016) assembled larger training sets by combining data from different generations and pools of a ryegrass breeding programme. The effect of training set size on PA was inconsistent and it was concluded that any benefit to PA from expanding the size of the training population may be confounded in part by genetic divergence amongst the different contributing groups. Ramstein et al. (2016) observed that pooling data from two small switchgrass breeding populations provided no benefit over single population genomic prediction models, and also attributed this to the extent of genetic divergence between the contributing populations. In flax, You et al. (2016) reported PA increased significantly when pooling certain biparental populations into training sets, but not all combinations. Population combinations that resulted in greater PA, compared with single population models, were not necessarily the largest and were most effective for low heritability traits. In the current dataset, a clear influence of genetic structure on the efficacy of the MP training set approach was difficult to discern. For example, PA was positive in Pop IV, despite the relative genetic divergence of this population from the other four in the training set. Overall, the increase in training set size achieved by combining populations may have been sufficient to mitigate any negative influence of genetic divergence amongst the individual populations.

Perennial forage species are generally bred for broad adaptation, as the market for cultivars adapted to specific environments is considered too small to justify development costs (Chapman et al. 2017). Genomic prediction models that predict mean DMY potential of genotypes across environments would, therefore, serve current industry breeding practice. For the MP training set, across-environment BLUP adjusted means for HA were successfully generated for only the combination of the two Ruakura environments (Rua STD + SEV) and of the two severe grazing treatments (Comb SEV),—specific cases where environments showed a degree of phenotypic correlation (Supplementary Figure S4, Table S2). Under standard grazing, pooling HA data for the MP training set from all three locations failed to resolve significant genotypic variation amongst families and combining HA data across all five environments (locations, treatment) was similarly unsuccessful. Our results suggest that developing a genomic prediction model for inference of HA across all New Zealand environments may be difficult to achieve due to substantial genotype-by-environment interaction and relatively high-pooled error variance (Table 2). An alternative approach to implementing genomic prediction for mean performance across environments may be to incorporate GEBV’s, estimated for a range of single-environment HA, into selection indices that weight HA across target environments (Jahufer and Casler 2015). However, the correlated response will depend on the type, positive or negative, and magnitude of the genetic correlation among the target environments.

Higher PA’s (*r* = 0.36, 0.43) were, nevertheless, achieved in the current study in two cases where data were successfully pooled across environments, Comb SEV and Rua STD + SEV. The higher PA that resulted from pooling data in these cases may be a consequence of underlying genetic correlation between environments, effectively increasing the number of measurements per genotype and the reliability of the HS-family BLUP’s for those traits, which has implications for the design of future training experiments that utilise these trial formats.

As described by others (Heffner et al. 2010; Resende et al. 2014), GS may be used to increase the rate of genetic gain per unit time by reducing the length of the generation interval. In forage species, Annicchiarico et al. (2015) illustrated that for DMY, which typically requires multi-year phenotypic assessment, GS using a prediction model with even modest PA has the potential to deliver more than three-fold increase in efficiency over a conventional scheme, by reducing the length of the generation interval from 3 years to 1. Additionally, by enabling the breeder to access the within-family ¾ additive genetic variation that is ordinarily difficult to leverage (Casler and Brummer 2008), GS also offers a means to increase the rate of genetic gain in a single cycle of selection. Simulation based on our experimental data showed that ∆*G*_c_ from a single cycle of selection for HA could be almost doubled relative to conventional HS family selection, using among-family selection based on phenotype in combination with within-family selection by GS with a moderate PA (*r* = 0.27) and high-selection intensity. In a practical breeding sense, 3 years of field evaluation could be used to phenotype and rank HS families, enabling selection of the best families. The phenotypic data generated in the field phase would also be used, in conjunction with maternal parent genotypic data, to generate a genomic prediction model that is then applied for the within-HS family selection component. Because GS occurs within the same generation used to train the prediction model, erosion of PA over more than one cycle of selection (see below) is not a factor in this situation.

Persistency of PA when applying a genomic prediction model long term, over successive cycles of selection, will be affected by the relative contributions to PA of three sources of information: pedigree relationships captured by SNPs; linkage between QTL and SNPs; or ancestral LD between QTL and SNPs (Habier et al. 2007, 2013; Müller et al. 2017). PA due to ancestral LD is expected to persist across generations while the impact of genetic relatedness, which decays rapidly over generations, has been shown to be the prevalent component of PA in GBLUP approaches in particular (Habier et al. 2007; Zhong et al. 2009). It is not possible to quantify the contributions of these different components to PA of prediction models developed with the current dataset. However, LD decayed rapidly in the individual populations of the training set, and therefore, LD underlying the MP training set as a whole would be expected to be low. Given the absence of long range LD and the observation that PA was unchanged despite a 25-fold reduction in SNP marker density (from 1.02 M to 44 K SNPs), it is reasonable to conclude that PA estimated for the MP genomic prediction models is predominantly due to the capture of genetic relationships between training and validation sets. A practical implication of this is that retraining of the genomic prediction models might be needed in each generation to fully exploit pedigree relationships, as this source of PA erodes rapidly over generations. However, some level of SNP-QTL LD may exist across the training set, given (a) the efficacy of the MP models across the full range of populations in the set and (b) the prevalent contribution of only three principal germplasm sources in New Zealand ryegrass breeding (Stewart 2006). Validation of longer term PA, by completing successive cycles of GS and progeny evaluation from training set-related and independent populations, will enable empirical evaluation of PA decay and the contribution of population-wide SNP QTL-LD to the genomic prediction models established in this study.

Only small differences in PA were found amongst the analytical approaches assessed, and there was no obvious interaction with trait type or repeatability/heritability. GBLUP and KGD, a modified GBLUP approach developed to deal with GBS genotypes associated with low-read depth and without imputing missing data, were most often equal to or slightly superior to another genomic linear method, RR while the non-parametric machine-learning method, RF ranked lowest overall. This may reflect conditions (limited size and multi-population composition of the training set) that favour the ability of GBLUP methods to capture genetic relationship information in the SNP dataset. Expansion to a larger training set, based on a single population, may identify differences in PA of different analytical approaches in ryegrass. This should include the application of Bayesian methods, which were not assessed here, but which were found to be superior to GBLUP in achieving high PAs when using a multi-breed training set (Hayes et al. 2009a).

We have demonstrated that a ryegrass training set composited from multiple breeding populations, in conjunction with genotyping-by-sequencing as a source of molecular marker information, provides an encouraging basis for tuning a GS engine to specific applications in terms of traits, locations and farm systems. At the scale of training used in this study, cross-validation prediction accuracies estimated for within and across-environment HA have the potential to deliver a significant enhancement of genetic gain inside a single cycle of selection, if used for within-HS family selection at relatively high selection intensity. Validation of PA via multiple selection cycles in independent populations is required to determine (a) model persistence and efficacy over more than one selection cycle from the training generation and (b) transferability of the constructed models to populations unrelated to the training set. Sown rows, used in this training experiment instead of larger plots, have been shown to be correlated with sward yield and persistence in large rectangular plots (1.5 m × 5 m) in a ryegrass breeding programme (Dr. M. Z. Z. Jahufer, unpublished data). Nevertheless, validation of the genomic prediction models described here will require that experimental synthetic developed by GS are assessed in larger plots and future genomic selection training systems should ideally use larger plot formats that more closely resemble on-farm swards.

### **Author contribution statement**

MF conceived the study, designed and coordinated the training experiment, completed statistical analysis of phenotypic data, contributed to development of GBS protocols and wrote the paper. SG evaluated and applied genomic prediction statistical models and imputation methods. MC developed an informatics pipeline for GBS data analysis and completed GBS data analysis for the training set. ZJ designed the field trials, statistically analysed phenotypic data and completed simulations. TB completed the linkage disequilibrium analysis. SE conceived the study, established field trials and statistically analysed phenotypic data. AG developed GBS protocols and RT co-developed and applied GBS protocols. RM compiled the ryegrass reference genome used for supporting GBS. BB wrote the grant and contributed to project execution. JS, CF, DR, JT and PR were in charge of field trials at different locations and acquired the phenotypic data. SG, MC, ZJ, TB, SE, AG, RM, PR and BB all contributed to writing the paper.

## Electronic supplementary material

Below is the link to the electronic supplementary material.

Supplementary material 1 (DOCX 1408 kb)
