# Supplementary material for: Predictive ability of genomic selection models in a multi-population perennial ryegrass training set using genotyping-by-sequencing
Source: Theor Appl Genet. 2017 Dec 20;131(3):703–20. doi: 10.1007/s00122-017-3030-1 (PMC5814531; doi:10.1007/s00122-017-3030-1)
Supplement: Supplementary file 1 — Supplementary material 1 (DOCX 1408 kb) [file 122_2017_3030_MOESM1_ESM.docx]

**Supplementary materials**

**Climate data**

During 2014 - 2016 the Lincoln trial site was the driest (mean annual rainfall 518 mm) of the three sites (Figure S5) and the coolest, experiencing the lowest mean air temperature annually as well as during the summer (December – February) (Figure S3). Ruakura had the highest mean annual rainfall (1033 mm) and, during the experimental timeframe, was only appreciably surpassed by Aorangi (annual mean 947 mm) during the autumn-winter periods (March – June) of 2015 and 2016 (Figure 3). Ruakura was the warmest site, characterised by mean air temperatures 0.8°C higher than Aorangi and 2.3°C more than Lincoln over the experimental period. Over the two the summer periods captured by this trial, Ruakura experienced more days (96) on which air temperature exceeded 25°C than both Aorangi (64 days) and Lincoln (47 days). Overall, there was greater similarity in climatic conditions between Ruakura and Aorangi, with the Lincoln site distinct from these.

**Figure S1.** Total monthly rainfall and mean monthly air temperature recorded for the Ruakura, Aorangi and Lincoln experimental sites during 2014 – 2016.


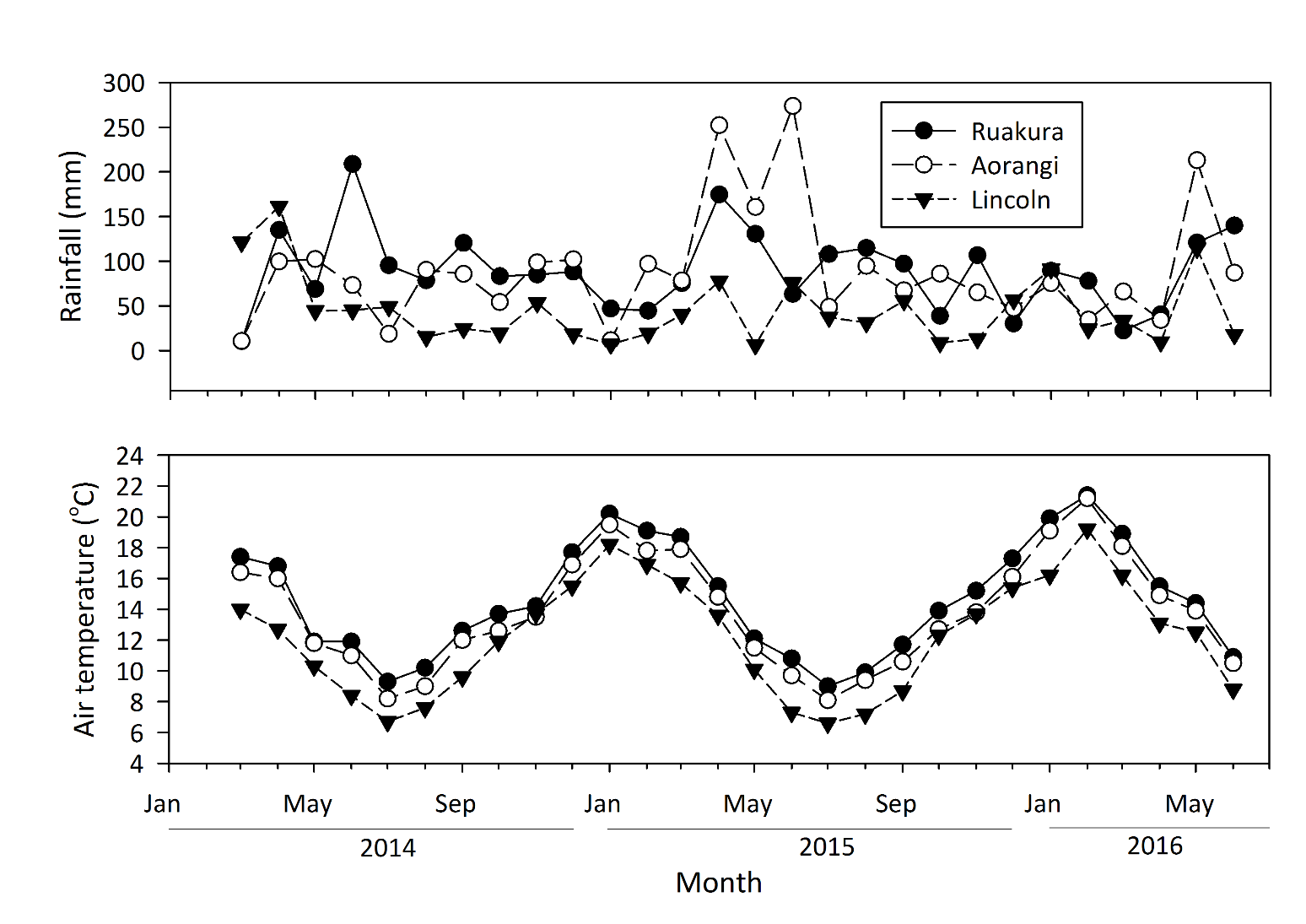


**Phenotyping methods**

**Population development**

Between 102 and 115 plants from each population (577 plants in total) that tested positive for endophyte infection by immunoblotting ([Hahn et al. 2003](#_ENREF_37)) were polycrossed under isolation during spring 2012 at Palmerston North, New Zealand. Discrete polycrosses were completed for each of Pop I to V (i.e. no mixing between populations). Maternal HS family seed was harvested from individual plants within each polycross. A total of 543 HS families were harvested and individually cleaned to achieve a thousand seed weight of approximately 1.8 g. Seed from 517 HS families was available in sufficient quantity (≥ 3.6g) for sowing in trials.

**Measurement of days-to-heading (DTH) – treatment of trials**

At the Lincoln site the trial was managed as a seed crop for the purposes of DTH measurement. The stem shortening plant growth regulator trinexapac-ethyl (Moddus) was applied as a split application and at high rates both to cover the range of flowering dates and to reduce lodging so rows were not shading neighbouring rows or becoming mixed. Moddus was applied at Zadoks growth stage 31-32 ([Zadoks et al. 1974](#_ENREF_84)) for early flowering material and growth stage 30-31 for late-flowering material, at 1.6 L/ha on 2 November 2015 with 0.6 l/ha of the fungicide Proline (250 g prothioconazole/L); and again on 18 November at 1.0 L/ha when early flowering rows were at growth stage 37-39 and late flowering at growth stage 31-32. At Ruakura no plant growth regulator was applied.

**Models used for statistical analysis of phenotypic data**

**Individual locations within treatments**

*Y_ijklmno_ = M + f_i_ + y_j_ + (fy)_ij_ + h_k_ + (fh)_ik_ + p_l_ +b_jkm_ + r_jkmn_ + c_jkmo_ + ε_ijklmno_*,

*Y_ijklmn_* is the value of an attribute measured from HS family *i* of population *l*, in row *n* and column *o* of replicate *m* nested in harvest *k* in year *j* and *i*=1,...,*n_f_*, *j*=1,...,*n_y_, k*=1,...,*n_h_, l*=1,...,*n_p_*, *m*=1,...,*n_b_,* *n*=1,...,*n_r_*, *o*=1,...,*n_c_*, where *f*, *y*, *h*, *p*, *b*, *r* and *c* are half sibling families, years, harvests, populations, replicates, rows and columns, respectively; *M* is the overall mean; *f_i_* is the random effect of HS family *i*, N(0,*σ^2^_f_*); *y_j_* is the fixed effect of year *j*; *(fy)_ij_* is the effect of the interaction between HS family *i* and year *j*, N(0,*σ^2^_fy_*); *h_k_* is the fixed effect of harvest *k*; *(fh)_ih_* is the effect of the interaction between HS family *i* and harvest *k*, N(0,*σ^2^_fh_*); p*_l_* is the fixed effect of population *l*; *b_jkm_* is the random effect of replicate *m* within harvest *k* in year *j* N(0,*σ^2^_b_*); *r_jklm_* is the random effect of row *n* within replicate *m* within harvest *k* in year *j*, N(0,*σ^2^_r_*); *c_jkmo_* is the random effect of column *o* within replicate *m* within harvest *k* in year *j*, N(0,*σ^2^_c_*); *ε_ijklmno_* is the residual effect of HS family *i* of population *l*, in row *n* and column *o* of replicate *m* during harvest *k* in year *j*, N(0,*σ^2^_ε_*).

**Individual locations across treatments**

*Y_ijklmnop_ = M + f_i_ + y_j_ + (fy)_ij_ + h_k_ + (fh)_ik_ + t_l_ +(ft)_il_ + p_m_ +b_jkln_ + r_jklno_ + c_jkinp_ + ε_ijklmnop_*,

*Y_ijklmnop_* is the value of an attribute measured from HS family *i* of population *m*, in row *o* and column *p* of replicate *n* nested in treatment *l* of harvest *k* in year *j* and *i*=1,...,*n_f_*, *j*=1,...,*n_y_, k*=1,...,*n_h_, l*=1,...,*n_t_*, *m*=1,...,*n_p_,* *n*=1,...,*n_b_*, *o*=1,...,*n_r_*, *p*=1,...,*n_c_*, where *f*, *y*, *h*, *t*, *p*, *b*, *r* and *c* are half sibling families, years, harvests, treatments, populations, replicates, rows and columns, respectively; *M* is the overall mean; *f_i_* is the random effect of HS family *i*, N(0,*σ^2^_f_*); *y_j_* is the fixed effect of year *j*; *(fy)_ij_* is the effect of the interaction between HS family *i* and year *j*, N(0,*σ^2^_fy_*); *h_k_* is the fixed effect of harvest *k*; *(fh)_ik_* is the effect of the interaction between HS family *i* and harvest *k*, N(0,*σ^2^_fh_*); *t_l_* is the fixed effect of treatment *l*; *(ft)_il_* is the effect of the interaction between HS family *i* and treatment *l*, N(0,*σ^2^_ft_*); *p_m_* is the fixed effect of population *m*; *b_jkln_* is the random effect of replicate *n* within treatment *l* of harvest *k* in year *j* N(0,*σ^2^_b_*); *r_jklno_* is the random effect of row *o* within replicate *n* within treatment *l* of harvest *k* in year *j*, N(0,*σ^2^_r_*); *c_jklnp_* is the random effect of column *o* within replicate *n* within treatment *l* of harvest *k* in year *j*, N(0,*σ^2^_c_*); *ε_ijklmnop_* is the residual effect of HS family *i* of population *m*, in row *o* and column *p* of replicate *n* in treatment *l* during harvest *k* in year *j*, N(0,*σ^2^_ε_*).

**Across locations within treatments**

*Y_ijklmnop_ = M + f_i_ + y_j_ + (fy)_ij_ + h_k_ + (fh)_ik_ + s_l_ +(fs)_il_ + p_m_ +b_jkln_ + r_jklno_ + c_jkinp_ + ε_ijklmnop_*,

*Y_ijklmnop_* is the value of an attribute measured from HS family *i* of population *m*, in row *o* and column *p* of replicate *n* nested in site *l* of harvest *k* in year *j* and *i*=1,...,*n_f_*, *j*=1,...,*n_y_, k*=1,...,*n_h_, l*=1,...,*n_s_*, *m*=1,...,*n_p_,* *n*=1,...,*n_b_*, *o*=1,...,*n_r_*, *p*=1,...,*n_c_*, where *f*, *y*, *h*, *s*, *p*, *b*, *r* and *c* are half sibling families, years, harvests, sites, populations, replicates, rows and columns, respectively; *M* is the overall mean; *f_i_* is the random effect of HS family *i*, N(0,*σ^2^_f_*); *y_j_* is the fixed effect of year *j*; *(fy)_ij_* is the effect of the interaction between HS family *i* and year *j*, N(0,*σ^2^_fy_*); *h_k_* is the fixed effect of harvest *k*; *(fh)_ik_* is the effect of the interaction between HS family *i* and harvest *k*, N(0,*σ^2^_fh_*); *s_l_* is the fixed effect of site *l*; *(fs)_il_* is the effect of the interaction between HS family *i* and site *l*, N(0,*σ^2^_fs_*); *p_m_* is the fixed effect of population *m*; *b_jkln_* is the random effect of replicate *n* within site *l* of harvest *k* in year *j* N(0,*σ^2^_b_*); *r_jklno_* is the random effect of row *o* within replicate *n* within site *l* of harvest *k* in year *j*, N(0,*σ^2^_r_*); *c_jklnp_* is the random effect of column *o* within replicate *n* within site *l* of harvest *k* in year *j*, N(0,*σ^2^_c_*); *ε_ijklmno_* is the residual effect of HS family *i* of population *m*, in row *o* and column *p* of replicate *n* in site *l* during harvest *k* in year *j*, N(0,*σ^2^_ε_*).

**Across sites and treatment**

*Y_ijklmnopq_ = M + f_i_ + y_j_ + (fy)_ij_ + h_k_ + (fh)_ik_ + s_l_ +(fs)_il_ + t_m_+ (ft)_im_ + p_n_ +b_jklmo_ + r_jklmop_ + c_jkimpq_ + ε_ijklmnopq_*,

*Y_ijklmnopq_* is the value of an attribute measured from HS family *i* of population *n*, in row *p* and column *q* of replicate *o* nested in site *l* of harvest *k* in treatment *m* during year *j* and *i*=1,...,*n_f_*, *j*=1,...,*n_y_, k*=1,...,*n_h_, l*=1,...,*n_s_*, *m*=1,...,*n_t_ n*=1,...,*n_p_,* *o*=1,...,*n_b_*, *p*=1,...,*n_r_*, *q*=1,...,*n_c_*, where *f*, *y*, *h*, *s*, *t*, *p*, *b*, *r* and *c* are half sibling families, years, harvests, sites, treatments, populations, replicates, rows and columns, respectively; *M* is the overall mean; *f_i_* is the random effect of HS family *i*, N(0,*σ^2^_f_*); *y_j_* is the fixed effect of year *j*; *(fy)_ij_* is the effect of the interaction between HS family *i* and year *j*, N(0,*σ^2^_fy_*); *h_k_* is the fixed effect of harvest *k*; *(fh)_ik_* is the effect of the interaction between HS family *i* and harvest *k*, N(0,*σ^2^_fh_*); *s_l_* is the fixed effect of site *l*; *(fs)_il_* is the effect of the interaction between HS family *i* and site *l*, N(0,*σ^2^_fs_*); *t_l_* is the fixed effect of treatment *m*; *(ft)_im_* is the effect of the interaction between HS family *i* and treatment *m*, N(0,*σ^2^_ft_*); *p_n_* is the fixed effect of population *n*; *b_jklmo_* is the random effect of replicate *o* within treatment *m* in site *l* of harvest *k* in year *j* N(0,*σ^2^_b_*); *r_jklmop_* is the random effect of row *p* within replicate *o* of treatment *m* in site *l* of harvest *k* in year *j*, N(0,*σ^2^_r_*); *c_jkimpq_* is the random effect of column *q* within replicate *o* of treatment *m* in site *l* of harvest *k* in year *j*, N(0,*σ^2^_c_*) N(0,*σ^2^_c_*); *ε_ijklmnoq_* is the residual effect of HS family *i* of population *n*, in row *p* and column *q* of replicate *o* of treatment *m* in site *l* during harvest *k* in year *j*, N(0,*σ^2^_ε_*).

**GBS data and LD analysis**

**Figure S2.** Distribution of minor allele frequency amongst 1,023,011 SNPs derived by genotyping-by-sequencing for a perennial ryegrass multi-population genomic selection training set.


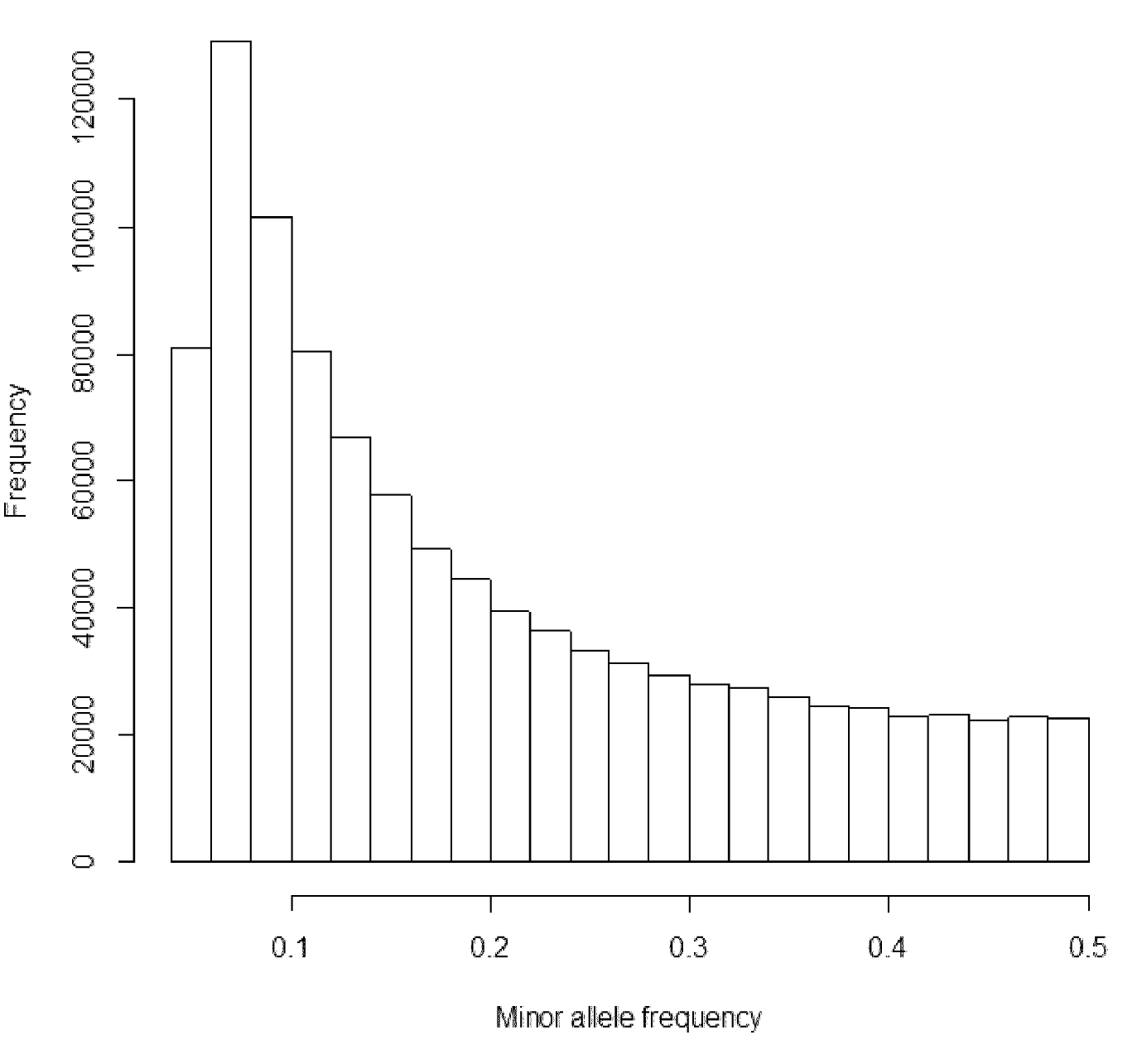


**Figure S3.** Hexbin plots of pairwise *r^2^* values between all SNPs mapped to the same scaffold, for all pseudo-chromosomes within each of Pop I – V. Each hexbin is coloured based on the number of points in the bin. The blue line is a non-linear regression model fitted to the data and the black line is the mean *r^2^* value across binned distances.


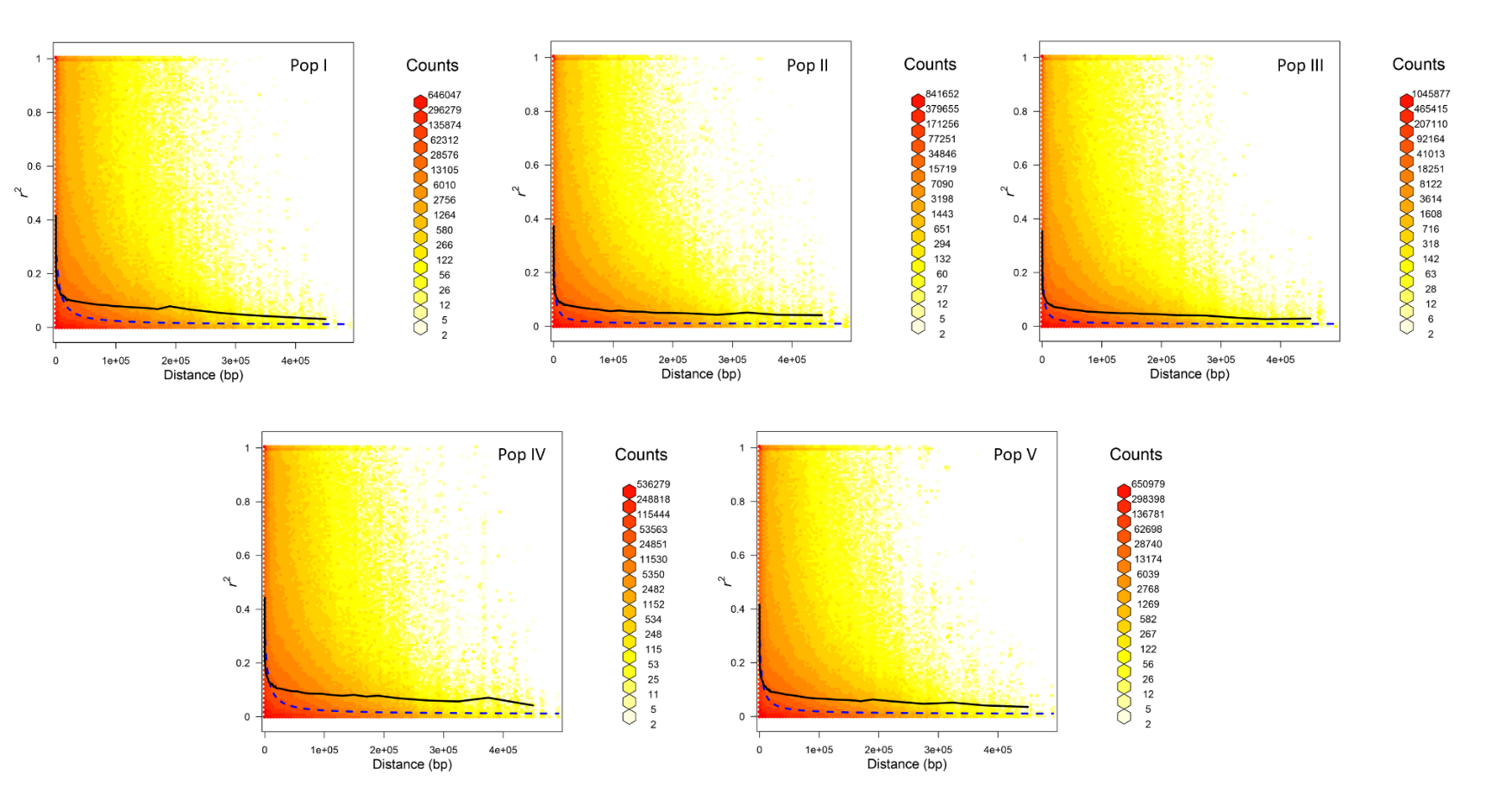


**Phenotypic data**

**Table S1.** Trial establishment and harvest dates, genotypic (σ^2^_g_) and experimental error (σ^2^_ε_) variance components and their associated standard errors (±SE) and repeatability (*R*) estimated for single-measure DMY in six environments. Italicised σ^2^_g_ figures indicate no statistically significant variation (*P* > 0.05) amongst HS families. STD = standard grazing management; SEV = severe summer grazing management; IRR = supplementary irrigation provided.

| **Site, Treatment** | **Year** | **Season** | **Measure** | **σ^2^_g_ ± SE** | **σ^2^_ε_ ± SE** | ***R*** |
| --- | --- | --- | --- | --- | --- | --- |
| Ruakura STD | 2014 | Summer | DM 0314 | 40.78 ± 4.42 | 61.56 ± 3.02 | 0.67 |
| (established April 2013) | 2014 | Autumn | DM 0614 | 7.48 ± 1.6 | 39.41 ± 1.89 | 0.36 |
|  | 2014 | Spring | DM 0914 | 7.72 ± 1.7 | 43.29 ± 2.07 | 0.35 |
|  | 2015 | Summer | DM 0215 | 12.63 ± 2.14 | 47.09 ± 2.27 | 0.45 |
|  | 2015 | Autumn | DM 0615 | 15.9 ± 4.4 | 127.5 ± 6 | 0.27 |
|  | 2015 | Spring | DM 0915 | 41.4 ± 6.4 | 130.7 ± 6.3 | 0.49 |
|  | 2016 | Summer | DM 0316 | 39.52 ± 5.34 | 96.9 ± 4.71 | 0.55 |
|  | 2016 | Autumn | DM 0516 | 39.5 ± 6.4 | 124.4 ± 6.4 | 0.49 |
| Ruakura SEV | 2014 | Summer | Insufficient growth | - | - | - |
| (established April 2013 | 2014 | Autumn | DM 0614 | 10.03 ± 1.91 | 49.12 ± 2.21 | 0.38 |
|  | 2014 | Spring | DM 0914 | 6.92 ± 2.19 | 71.75 ± 3.17 | 0.22 |
|  | 2014 | Spring | DM 1114 | 25.5 ± 5.3 | 141.4 ± 6.4 | 0.35 |
|  | 2015 | Summer | DM 0215 | *-1.02 ± 0.84* | 52.62 ± 2.25 | - |
|  | 2015 | Autumn | DM 0615 | 23.95 ± 4.03 | 94.73 ± 4.32 | 0.43 |
|  | 2015 | Spring | DM 0915 | 29.3 ± 5.9 | 151.5 ± 6.9 | 0.37 |
|  | 2016 | Summer | Insufficient growth | - | - | - |
|  | 2016 | Autumn | Trial damaged by livestock | - | - | - |
| Aorangi STD | 2014 | Summer | Insufficient growth | - | - | - |
| (established October 2013) | 2014 | Autumn | DM 0514 | 41.7 ± 9.3 | 234.4 ± 11.3 | 0.35 |
|  | 2014 | Spring | DM 1014 | *-0.30 ± 2.7* | 138.8 ± 6.1 | - |
|  | 2015 | Summer | DM 0215 | 25.9 ± 4.42 | 96.73 ± 4.67 | 0.45 |
|  | 2015 | Autumn | DM 0515 | 1.89 ± 0.58 | 17.18 ± 0.81 | 0.25 |
|  | 2015 | Spring | DM 0915 | 18.2 ± 6.4 | 196.5 ± 9.3 | 0.22 |
|  | 2015 | Spring | DM 1115 | *11.7 ± 8.2* | 308.8 ± 14.2 | - |
|  | 2016 | Summer | DM 0316 | 2.89 ± 1.43 | 50.78 ± 2.35 | 0.15 |
|  | 2016 | Autumn | DM 0516 | *3.18 ± 2.13* | 79.43 ± 3.66 | - |
| Aorangi SEV | 2014 | Summer | DM0314 | *0.38 ± 0.46* | 18.41± 0.83 | - |
| (established September 2013) | 2014 | Autumn | Insufficient growth | - | - | - |
|  | 2014 | Spring | DM 1014 | 10.5 ± 5.1 | 223.8 ± 10.1 | 0.12 |
|  | 2015 | Summer | Insufficient growth | - | - | - |
|  | 2015 | Autumn | DM 0515 | 1.98 ± 0.44 | 11.77 ± 0.54 | 0.34 |
|  | 2015 | Spring | DM 0915 | 23.9 ± 5.7 | 157.7 ± 7.2 | 0.31 |
|  | 2015 | Spring | DM 1115 | 41.7 ± 10.5 | 298.9 ± 13.7 | 0.30 |
|  | 2016 | Summer | Insufficient growth | - | - | - |
|  | 2016 | Autumn | DM 0516 | 17.32 ± 3.03 | 71.15 ± 3.31 | 0.42 |
| Lincoln STD | 2014 | Summer | DM 0314 | 62.7 ± 8.3 | 135.5 ± 6.9 | 0.58 |
| (established May 2013) | 2014 | Autumn | DM 0514 | *1.52 ± 2.06* | 76.52 ± 3.66 | - |
|  | 2014 | Spring | DM 1014 | 3.32 ± 1.25 | 36.77 ± 1.81 | 0.21 |
|  | 2015 | Summer | Insufficient growth | - | - | - |
|  | 2015 | Autumn | DM 0515 | *2.7 ± 5.1* | 187 ± 9.1 | - |
|  | 2015 | Spring | DM 0915 | 38.8 ± 18.5 | 570.3 ± 28.2 | 0.17 |
|  | 2015 | Spring | DM 1115 | 13.67 ± 3.49 | 88.28 ± 4.43 | 0.32 |
|  | 2016 | Summer | DM 0216 | 13.2 ± 6.1 | 228.3 ± 11.1 | 0.15 |
|  | 2016 | Autumn | DM 0516 | *8.0 ± 8.1* | 287.8 ± 13.8 | - |
| Lincoln IRR | 2014 | Summer | DM 0314 | *3.6 ± 6.1* | 228.2 ± 11.3 | - |
| (established May 2013) | 2014 | Autumn | DM 0514 | *-0.02 ± 2.87* | 94.17 ± 5.51 | - |
|  | 2014 | Spring | DM 1014 | *5.82 ± 3.16* | 99.17 ± 5.03 | - |
|  | 2015 | Summer | DM 0215 | *28.1 ± 15.6* | 486.7 ± 24.8 | - |
|  | 2015 | Autumn | DM 0515 | *-0.6 ± 1.46* | 61.4 ± 3 | - |
|  | 2015 | Spring | DM 0915 | 24.1 ± 6.7 | 168.7 ± 8.7 | 0.30 |
|  | 2016 | Summer | Insufficient growth | - | - | - |
|  | 2016 | Autumn | DM 0516 | *1.54 ± 1.7* | 57.69 ± 2.91 | - |

**Description of variance component analysis of HA and Comb DTH data**

The trait Aor STD+SEV showed no significant (*P* > 0.05) σ^2^_g_, for either the MP training set or individual populations, and so no data are presented. For the most part there was no significant (*P* > 0.05) between genotype-by-harvest interaction (σ^2^_gh_), with the exception of Rua STD, Comb SEV and Aor SEV, the highest magnitude of which was observed for the latter. Significant (*P* < 0.05*)* genotype-by-year interaction (σ^2^_gy_) was also observed and was most clearly influential for the Rua STD trial (Table 2), the largest and most complete seasonal dataset amongst the five trials (n=8 single HA harvests over the experimental period).

HS family mean *R*, which estimates the upper limit of heritability, varied for HA traits in the MP training set (Table 2). Greater *R* or *h^2^_n_* for the two severe grazing treatment trials was driven principally by σ^2^_g or_ σ^2^_a_ that was substantially higher than that observed for their standard treatment counterparts, whilst σ^2^_ε_ was similar between these treatment pairs (Table 2). *R* or *h^2^_n_* for HA traits that combined data across locations or treatments were lower than those estimated within a location or treatment, with significant genotype-by-environment effects (σ^2^_gl_ and σ^2^_gt_) contributing. Amongst the individual populations Pop II was the highest performer for DMY at Ruakura and under severe grazing at Aorangi, while Pop V was more productive under standard grazing at the Aorangi and Lincoln sites (Table 2). Comb DTH σ^2^_gl_ was significant (*P* <0.05) for the MP training set and one individual population but the relative magnitude of this interaction was lower than that observed for HA traits. *R* and *h^2^_n_* were moderate to high for Comb DTH (Table 2).

**Phenotypic correlation amongst environments**

Phenotypic correlations (Table S2) for HA were moderate and significant (*P* < 0.05) within locations, between Rua STD and SEV (*r* = 0.47) and Aor STD and SEV (*r* = 0.33). Between locations, Aor SEV was significantly correlated with both Rua SEV (*r* = 0.37) and Rua STD (*r* = 0.34). Weaker but significant (*P* > 0.05) correlations occurred between Aor STD and Rua STD and Rua SEV (both *r* = 0.24). Lin STD did not correlate significantly (*P* > 0.05) with any other trial environment. These associations were reflected in a cluster analysis of the five environments, based on HA data from all HS families, with HA performance clearly delineated based on location and grazing treatment (Figure S6). Ruakura, Aorangi and Lincoln each formed discrete groups, with a closer association between the two more climatically-similar North island sites. Within both the Ruakura and Aorangi locations there was further separation based on trial treatment (standard vs severe summer grazing).

**Table S2.** Phenotypic correlation (*r*) among environments based on mean herbage accumulation (HA). Note that the original matrix used in cluster analysis consisted of all the 517 HS families representing five populations. However, to conduct the phenotype correlation analysis the number of HS families was reduced to 435 some families having missing values in some environments.

|  | Aor SEV | Aor STD | Lin STD | Rua SEV |
| --- | --- | --- | --- | --- |
| Aor STD | 0.33^**^ |  |  |  |
| Lin STD | 0.06^ns^ | 0.11^ns^ |  |  |
| Rua SEV | 0.37^**^ | 0.24^**^ | 0.19^**^ |  |
| Rua STD | 0.34^**^ | 0.25^**^ | 0.18^**^ | 0.47** |

**Figure S4.** Dendrogram based on cluster analysis of the 517 HS family-by-five environment (Aor STD, Aor SEV, Rua STD, Rua SEV and Lin STD) matrix of Best linear Unbiased Predicted means (BLUP) for herbage accumulation (HA).


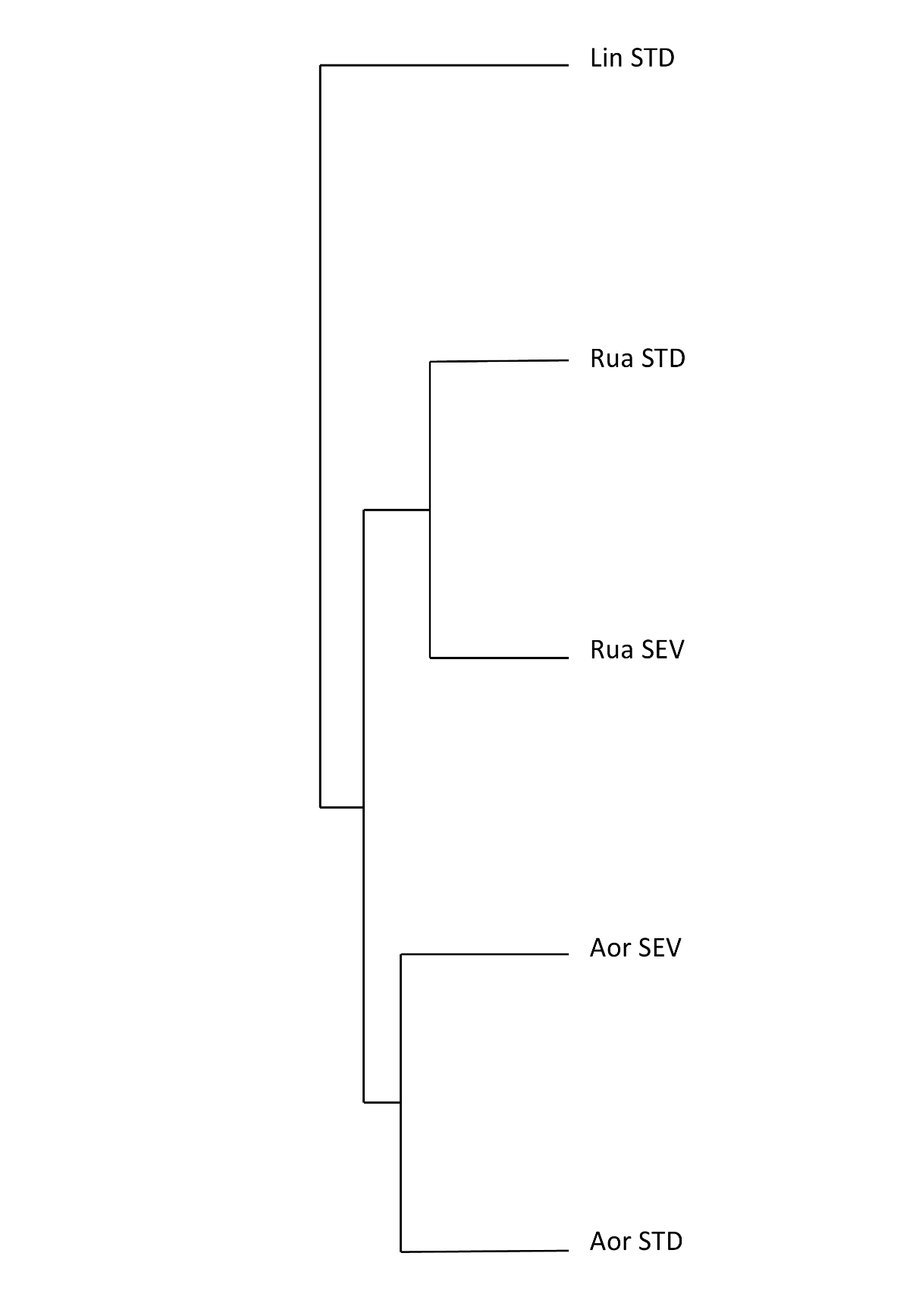


**Genomic prediction**

**Figure S5.** Plots of mean genomic-estimated breeding values, generated from ten repeats of 10-fold cross-validation (10CV_10rpt) compared with five repeats (10CV_5rpt), for six traits using the KGD method.


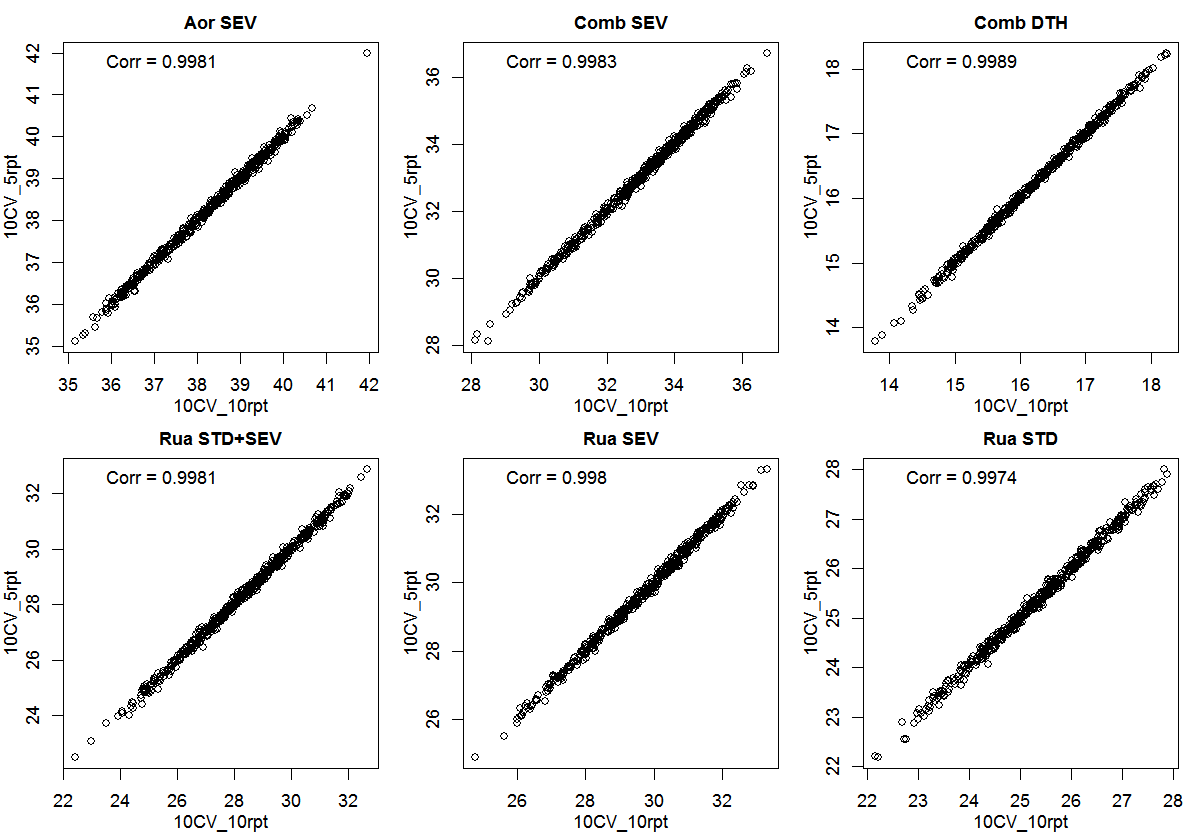


**Figure S6.** Cross-validation (CV) predictive ability for five herbage accumulation traits and days-to-heading (Comb DTH), comparing mean PA from five repeats of 10-fold CV (blue), with ten repeats (red). Error bars are standard error.


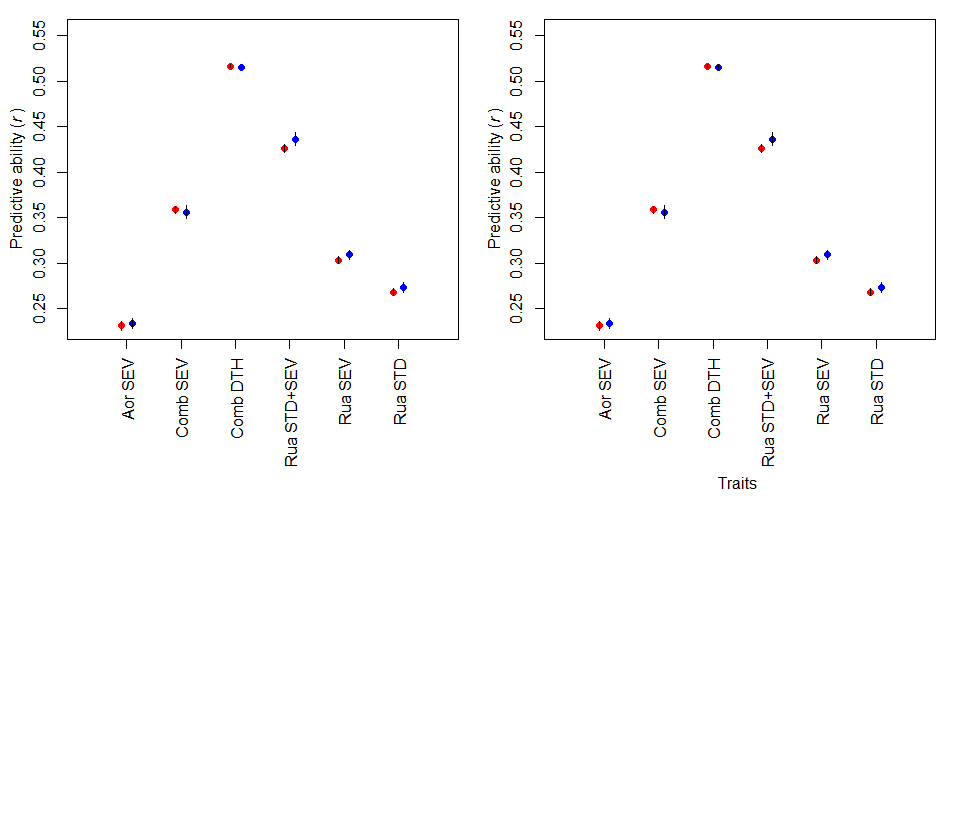


**Table S3.** Effect of statistical model (KGD = KGD-GBLUP; RR = Ridge Regression; RF = Random Forest), SNP missing data rate and minimum SNP read depth on mean (n = 5) 10-fold cross validation predictive ability (*r*). Data is shown for seven herbage accumulation traits and days-to-heading (Comb DTH). Standard error is in brackets.

| Method | Read depth | Missing data (%) | No. SNPs | Aor STD | Aor SEV | Lin STD | Rua STD | Rua SEV | Rua  STD+ SEV | Comb SEV | Comb DTH |
| --- | --- | --- | --- | --- | --- | --- | --- | --- | --- | --- | --- |
| KGD | >1 | 50 | 1023011 | 0.22 (0.005) | 0.24 (0.004) | 0.10 (0.011) | 0.26 (0.005) | 0.30 (0.007) | 0.43 (0.006) | 0.36 (0.006) | 0.52 (0.005) |
| RR | >1 | 1 | 43996 | 0.23 (0.007) | 0.21 (0.005) | 0.13 (0.006) | 0.28 (0.006) | 0.28 (0.010) | 0.43 (0.007) | 0.33 (0.011) | 0.51 (0.002) |
| RR | >1 | 10 | 249546 | 0.19 (0.007) | 0.23 (0.005) | 0.15 (0.006) | 0.30 (0.007) | 0.30 (0.008) | 0.44 (0.008) | 0.35 (0.010) | 0.51 (0.003) |
| RR | >7 | 1 | 58952 | 0.22 (0.008) | 0.24 (0.003) | 0.09 (0.004) | 0.31 (0.006) | 0.28 (0.009) | 0.43 (0.008) | 0.34 (0.008) | 0.53 (0.002) |
| RR | >7 | 10 | 88688 | 0.25 (0.008) | 0.21 (0.005) | 0.10 (0.005) | 0.30 (0.004) | 0.28 (0.010) | 0.43 (0.007) | 0.33 (0.008) | 0.52 (0.003) |
| RR | >7 | 50 | 93560 | 0.25 (0.009) | 0.21 (0.003) | 0.11 (0.005) | 0.30 (0.004) | 0.29 (0.010) | 0.43 (0.007) | 0.33 (0.008) | 0.46 (0.004) |
| RF | >1 | 1 | 43996 | 0.23 (0.007) | 0.26 (0.004) | 0.11 (0.006) | 0.25 (0.007) | 0.32 (0.007) | 0.40 (0.006) | 0.36 (0.005) | 0.47 (0.004) |
| RF | >1 | 10 | 249546 | 0.23 (0.008) | 0.26 (0.003) | 0.14 (0.005) | 0.25 (0.007) | 0.31 (0.006) | 0.40 (0.005) | 0.35 (0.006) | 0.47 (0.004) |
| RF | >1 | 50 | 1023011 | 0.23 (0.003) | 0.24 (0.005) | 0.15 (0.010) | 0.24 (0.004) | 0.30 (0.006) | 0.39 (0.004) | 0.34 (0.005) | 0.46 (0.005) |
| RF | >7 | 1 | 58952 | 0.22 (0.008) | 0.26 (0.006) | 0.07 (0.008) | 0.24 (0.011) | 0.30 (0.005) | 0.38 (0.007) | 0.35 (0.010) | 0.46 (0.004) |
| RF | >7 | 10 | 88688 | 0.23 (0.009) | 0.26 (0.005) | 0.09 (0.006) | 0.27 (0.007) | 0.29 (0.004) | 0.39 (0.007) | 0.34 (0.006) | 0.46 (0.004) |
| RF | >7 | 50 | 93560 | 0.23 (0.005) | 0.25 (0.002) | 0.13 (0.011) | 0.26 (0.003) | 0.30 (0.007) | 0.39 (0.005) | 0.34 (0.006) | 0.46 (0.003) |
| GBLUP | >1 | 1 | 43996 | 0.23 (0.006) | 0.24 (0.006) | 0.10 (0.003) | 0.27 (0.005) | 0.31 (0.006) | 0.43 (0.006) | 0.35 (0.007) | 0.52 (0.002) |
| GBLUP | >1 | 10 | 249546 | 0.22 (0.006) | 0.24 (0.006) | 0.10 (0.003) | 0.27 (0.006) | 0.31 (0.005) | 0.44 (0.007) | 0.36 (0.007) | 0.52 (0.003) |
| GBLUP | >1 | 50 | 1023011 | 0.21 (0.006) | 0.23 (0.006) | 0.07 (0.009) | 0.27 (0.006) | 0.31 (0.005) | 0.44 (0.007) | 0.36 (0.007) | 0.52 (0.003) |
| GBLUP | >7 | 1 | 58952 | 0.25 (0.007) | 0.24 (0.007) | 0.07 (0.003) | 0.28 (0.004) | 0.29 (0.006) | 0.43 (0.007) | 0.35 (0.006) | 0.52 (0.002) |
| GBLUP | >7 | 10 | 88688 | 0.25 (0.007) | 0.23 (0.007) | 0.07 (0.004) | 0.28 (0.004) | 0.30 (0.006) | 0.43 (0.007) | 0.35 (0.007) | 0.52 (0.003) |
| GBLUP | >7 | 50 | 93560 | 0.25 (0.007) | 0.23 (0.007) | 0.08 (0.004) | 0.28 (0.004) | 0.30 (0.007) | 0.43 (0.007) | 0.35 (0.007) | 0.52 (0.003) |
